# Genome analysis of *Paenibacillus polymyxa* A18 gives insights into the features associated with its adaptation to the termite gut environment

**DOI:** 10.1038/s41598-019-42572-5

**Published:** 2019-04-15

**Authors:** Nandita Pasari, Mayank Gupta, Danish Eqbal, Syed Shams Yazdani

**Affiliations:** 1 Microbial Engineering Group, International Centre for Genetic Engineering and Biotechnology, New Delhi, India; 2 DBT-ICGEB Centre for Advanced Bioenergy Research, International Centre for Genetic Engineering and Biotechnology, New Delhi, India

## Abstract

*Paenibacillus polymyxa* A18 was isolated from termite gut and was identified as a potential cellulase and hemicellulase producer in our previous study. Considering that members belonging to genus *Paenibacillus* are mostly free-living in soil, we investigated here the essential genetic features that helped *P. polymyxa* A18 to survive in gut environment. Genome sequencing and analysis identified 4608 coding sequences along with several elements of horizontal gene transfer, insertion sequences, transposases and integrated phages, which add to its genetic diversity. Many genes coding for carbohydrate-active enzymes, including the enzymes responsible for woody biomass hydrolysis in termite gut, were identified in *P. polymyxa* A18 genome. Further, a series of proteins conferring resistance to 11 antibiotics and responsible for production of 4 antibiotics were also found to be encoded, indicating selective advantage for growth and colonization in the gut environment. To further identify genomic regions unique to this strain, a BLAST-based comparative analysis with the sequenced genomes of 47 members belonging to genus *Paenibacillus* was carried out. Unique regions coding for nucleic acid modifying enzymes like CRISPR/Cas and Type I Restriction-Modification enzymes were identified in *P. polymyxa* A18 genome suggesting the presence of defense mechanism to combat viral infections in the gut. In addition, genes responsible for the formation of biofilms, such as Type IV pili and adhesins, which might be assisting *P. polymyxa* A18 in colonizing the gut were also identified in its genome. *In situ* colonization experiment further confirmed the ability of *P. polymyxa* A18 to colonize the gut of termite.

## Introduction

Plant biomass or lignocellulosic biomass, which is composed of cellulose, hemicellulose, lignin, and pectin, is one of the largest repositories of naturally fixed carbon. In its natural form, plant biomass is highly recalcitrant and requires an array of enzymes for degradation of all its components^1^. Termites have the ability to hydrolyze lignocellulosic biomass particularly in the form of wood and hence mobilize the carbon reservoir within the biosphere^2^. For efficient hydrolysis of biomass, termites harbor a plethora of microorganisms in its hindgut that aids in its digestion^3^. Previous reports have shown immense diversity of microbes in its gut; with a report suggesting the presence of 216 different phylotypes^4^. Also, Tokuda G. and Watanabe H. have reported that gut sanitized termites are incapable of digesting the lignocellulosic biomass completely^5^. Since termite guts have a dense microbial flora actively involved in lignocellulose degradation, it has also served as an excellent platform for isolation of cellulolytic organisms. Screening of cellulolytic microorganisms from the termite gut in our previous study led to the identification of a strain designated A18, which was found to be the maximum producer of cellulases amongst all the isolates^6^. Characterization of A18 indicated that it belonged to species *Paenibacillus polymyxa*. Amongst all the gut isolates, its secretome possessed not only maximum biomass hydrolyzing capabilities but also maximal cellulase, mannanase, xylanase and glucanase activities^6^.

Strains of the *P. polymyxa* have been identified to be cellulolytic in nature but only a few have been reported to be isolated from insect gut. Previously, *P. polymyxa* ICGEB2008 was isolated in our laboratory from the cotton bollworm gut, which has shown potential for both biomass hydrolysis as well as the production of biofuel molecules^7,8^. Other strains of *P. polymyxa* like M1, SC2, CR1, Sb3-1 have been found to be cellulolytic in nature but all are present in the rhizosphere^9,10^. These species are known as plant growth promoting rhizobacterium (PGPR) and are used in agriculture and industries^11^. Moreover, other members of the genus *Paenibacillus* like *Paenibacillus* sp. JDR-2, *P. terrae* HPL-003, *P. mucilaginous* KNP414 are cellulolytic in nature but they are also present in either soil or plant rhizosphere^12,13^. Since most of the *Paenibacillus* species are found in plant rhizospheres and soil as free-living it was possible that the termite picked-up *P. polymyxa* A18 from the surroundings and it was selected during our screening process as a potential cellulase and hemicellulase producer. It was thus intriguing to determine if it had inherited any features to survive and colonize in the gut environment.

Microbes capable of residing in the gut are able to form a biofilm, which enables them to adhere to the gut lining^14,15^. Various proteins participating in biofilm synthesis, like capsular polysaccharides, RTX proteins, Type IV pili, Type 6 secretion systems, and adhesins, have been found to be associated with gut colonization^16,17^. These features present in gut symbionts allow the formation of mutualistic interactions with the host organism^18^. Also, it has been found that microbes present in the gut environment are prone to viral attack^18^. Organisms residing in the gut have evolved with defense mechanisms to face the viral threats. Restriction-modification systems and CRISPR elements that degrade foreign DNA have been identified earlier in the genomes of gut symbionts of insects^19^.

The gene repertoires of the genus *Paenibacillus* are in incessant flux and the genome size of *Paenibacillus* shows high plasticity^20,21^. In this study, we describe the draft genome assembly of *P. polymyxa* A18. In order to explore its biomass-degrading capability, carbohydrate-active enzymes (CAZymes) were identified amongst the genes predicted. The features unique to this genome were identified by comparing its genome to other members of the genus *Paenibacillus*. Its ability to colonize the termite gut was then established by performing *in situ* experiment.

## Results

### Insight into the genome of *P. polymyxa* A18 to evaluate its genetic diversity

**Figure 1:**
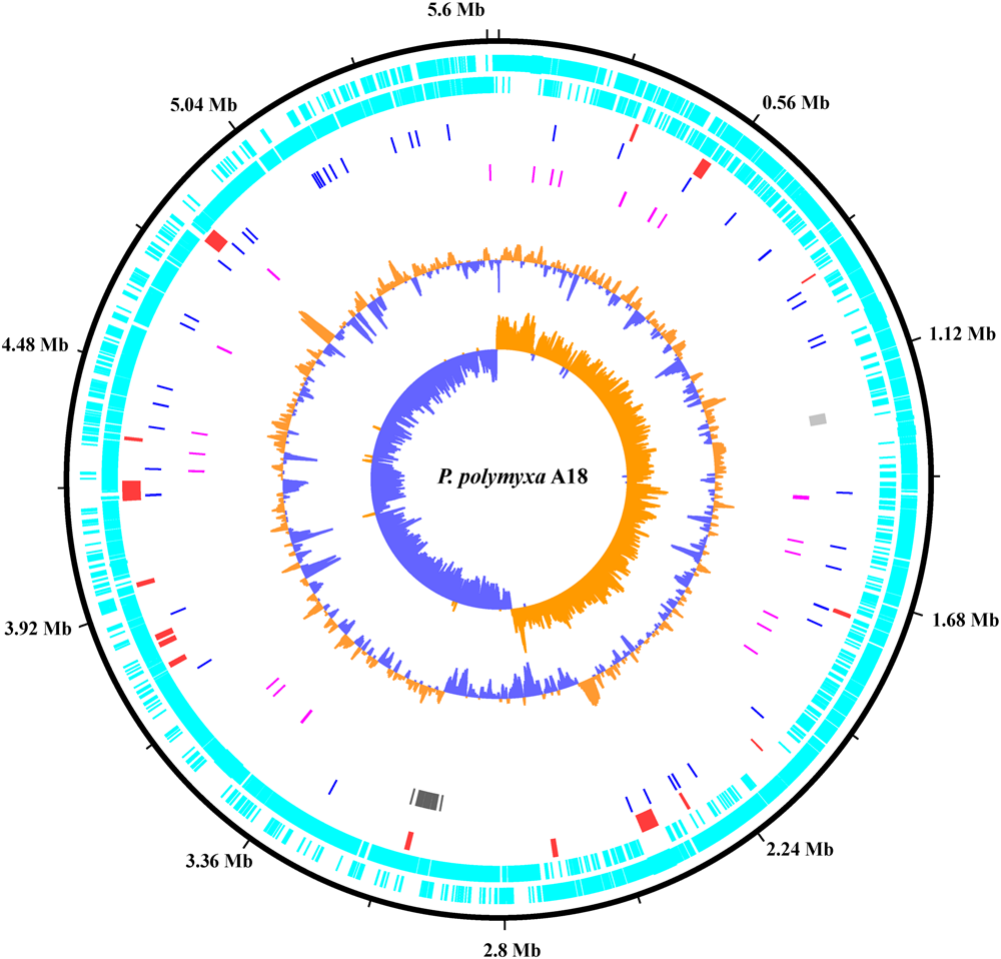
Circular map of *Paenibacillus polymyxa* A18 genome. From outer circle to inner circle, representation is as follows: 1. Position in megabases (black); 2. Forward strand CDSs (turquoise blue); 3. Reverse strand CDSs (turquoise blue); 4. Horizontal gene transfer (HGT) regions (red); 5. Insertion sequences (IS) (blue); 6. Phage sequences (grey); 7. tRNAs (pink); 8. GC plot (mustard and blue colour correspond to higher and lower than average GC content, respectively); 9. GC skew (mustard and blue colour correspond to higher and lower than average GC-skew, respectively).

*P. polymyxa* A18 was isolated from the gut of termite *Odontotermes hainanensis*^6^. In a quest to determine the distinguishing genomic features, its genome was sequenced using the GS-FLX Titanium platform. An FLX shotgun library and an 8-kb paired-end library were prepared from the genomic DNA sample. The shotgun library of 2,43,839 reads was assembled using GS Assembler, resulting in 682 contigs with a sequencing depth of 16.5-fold coverage. These contigs were joined using the 8-kb paired-end library into 28 scaffolds representing 5.72 Mb of the genome (Supplementary Table S1). The scaffolds were aligned with the reference genome of *P. polymyxa* M1 using CONTIGuator to obtain a single circularized genome (Fig. 1). The G + C content was predicted to be 46.2% with GC skew indicating the DNA leading strand, lagging strand, a replication origin, and replication terminal. tRNA scan predicted 105 tRNAs in the *P. Polymyxa* A18 genome (Supplementary Table S1).

Genes were predicted in the *P. polymyxa* A18 genome using Rapid Annotations using Subsystems Technology (RAST) Server (http://rast.nmpdr.org/)^22^ as well as National Center for Biotechnology Information (NCBI) prokaryotic annotation pipeline. RAST server predicted 5,059 CDS of which 1284 encoded for hypothetical proteins whereas NCBI based prediction accounted for 4608 CDS of which 987 were hypothetical (Additional file 1). 4608 CDS were found to be common amongst both the annotations, of which 366 were found to contain a secretory signal peptide, 1124 were predicted to contain transmembrane helices and 111 proteins were found to contain both secretory signal peptide as well as a transmembrane helix (Additional file1). Amongst the genes encoding for transmembrane proteins, proteins with functions of a transporter, sensor, flagellar or pilus proteins were detected.

The genetic diversity acquired by *P. polymyxa* A18 was identified by the genomic islands (GIs) it has incorporated in its genome in the form of horizontal gene transfers (HGTs) from distantly related organisms^23^. Foreign DNA acquired through HGT is generally associated with an anomalous GC content, insertion sequence (IS) elements, tRNA genes, and transposons. HGTs in *P. polymyxa* A18 genome were identified using the Island Viewer software^24^. A total of 17 genome islands were identified to have been acquired as HGTs from distantly related organisms (Supplementary Table S2). Many of the genes encoding for transporters and transcription factors were found in these HGTs. Entire stretches encoding for CRISPR associated proteins and Type-I restriction modification system were predicted to be acquired through HGT. Many genes in the stretches acquired as HGT encoded for proteins of unknown or hypothetical function (Supplementary Table S2).

Insertion elements, transposases, and integrated phages, which add to genetic diversity were also identified in *P. polymyxa* A18 genome^25^. Insertion sequences were identified using IS Finder and their genome locations are represented in Fig. 1. *P. polymyxa* A18 harbors 8 transposase containing IS elements. Not only can IS mobilize within a genome but it can also mobilize genes between bacterial strains or species allowing horizontal gene transfers^26^. Phages are another important vehicles for horizontal gene exchange between different bacterial species and account for a good share of the strain to strain differences within the same bacterial species^27^. Many prophages were detected in the genome suggesting that *P. polymyxa* A18 is a common phage target. Two major phage regions were detected in the genome with 23 phage insertions in region 1 (1231842-1255616) and 38 phage insertions in region 2 (2971359-3053311). Recombination sites *att*L and *att*R were found in region 2. The genetic diversity acquired by *P. polymyxa* A18 suggests that it is under the constant pressure of selection which is enabling it to adapt to the current environment.

### Phylogenetic analysis and genome alignment of *P. polymyxa* A18

The phylogenetic relationship was determined between *P. polymyxa* A18 and other completely sequenced members of *Paenibacillus* genus available at the NCBI server. General features of the *Paenibacillus* strains used for analysis are presented in Supplementary Table S3. As is evident, there is significant diversity amongst the *Paenibacillus* species with their genome size varying from 4.05 Mb in *Paenibacillus larvae* to 8.66 Mb *Paenibacillus mucilaginous* KNP414. With an increase in the genome size, the number of genes increased in these genomes. All *Paenibacillus* genomes analyzed here displayed an average GC content of 47.8%, which is consistent with the range of G + C mol% content of genomes of the *Paenibacillus* genus^10^.

**Figure 2:**
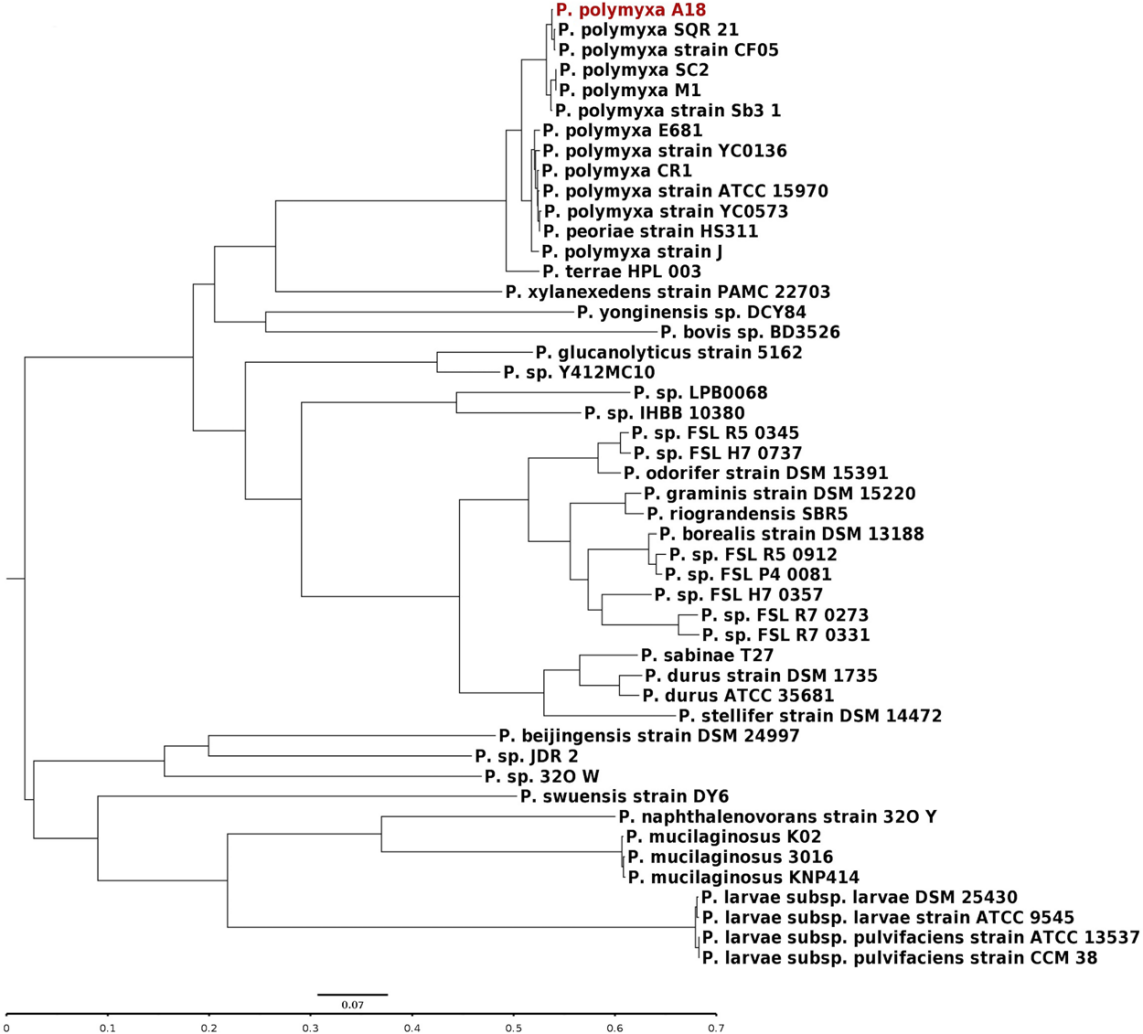
Phylogenetic analyses of the members of the genus *Paenibacillus*. Maximum-likelihood phylogenetic tree computed using the core genes of completely sequenced *Paenibacillus* sp. generated with 1000 bootstrap replications. Scale bar indicates the average number of substitutions per site.

**Figure 3:**
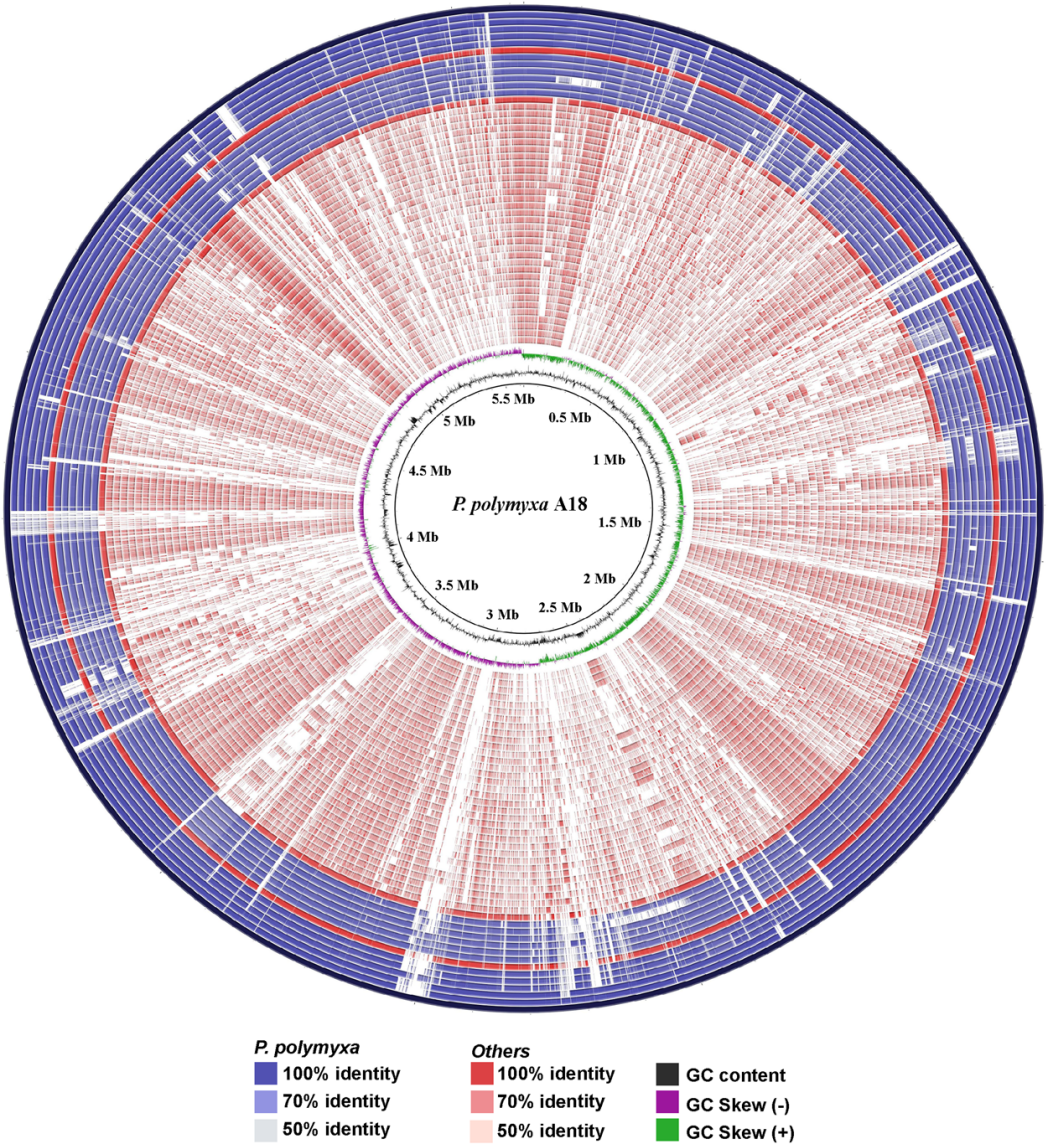
Comparative genome analyses of the members of the genus *Paenibacillus*. *P. polymyxa* A18 genome comparison with completely sequenced members of the genus *Paenibacillus*. Each of the genome sequence assemblies (the GenBank accession numbers are listed in Supplementary Table S3 in the order of their presence in the concentric rings) were aligned against the *P. polymyxa* A18 genome. The innermost ring indicates the genomic position. The next two rings represent G + C content and GC skew. The remaining concentric rings indicate the presence or absence of BLASTN hits at that position, with each ring corresponding to the genome assemblies in the order mentioned in Supplementary Table S3. Positions covered by BLASTN alignments are indicated with a solid color; white gaps represent genomic regions not covered by the BLASTN alignments. The graphical view of the alignments was rendered using BLAST Ring Image Generator (BRIG).

Coding sequences for 468 core genes were concatenated for construction of the phylogenetic tree based on the Maximum-likelihood method in RAxML8 (Fig. 2). The phylogenetic tree suggested that different species of *Paenibacillus* belonged to different clades and all strains of *P. polymyxa* were found to be clustered in one clad. *P. peoriae* HS311 was found to be present in the same clade as that of *P. polymyxa* suggesting a similarity to this species (Fig. 2). To glean global information into the nucleotide level differences, *P. polymyxa* A18 genome was used as reference sequence for alignment to the rest of the 47 completely sequenced *Paenibacillus* genomes (Supplementary Table S3) using BLAST Ring Image Generator (BRIG) (Fig. 3). The degree of alignment as displayed in Fig. 3 depicted that *P. polymyxa* A18 genome sequence exhibited more than 70% identity to the genome sequence of all the strains of *P. polymyxa*. Intriguingly, *P. peoriae* HS311 and *P. terrae* HPL-003 strains showed more than 70% similarity to the polymyxa species of *Paenibacillus*. This was in accordance with the phylogenetic tree which depicted that all the strains of *P. polymyxa* belonged to one cluster along with *P. peoriae* HS311 and *P. terrae* HPL-003 (Fig. 2). With rest of the species of *Paenibacillus*, *P. polymyxa* A18 showed rather a distant relationship, having much less than 70% identity (Fig. 3). Throughout the alignment, certain genomic regions of all *Paenibacillus* sp. were partially or completely absent when compared with *P. polymyxa* A18 genome. These regions could be associated with the unique features of *P. polymyxa* A18. These unique features along with some of the common features associated with *Paenibacillus* sp. that may help *P. polymyxa* A18 to live and colonize in the termite gut were explored further.

### Repertoire of CAZymes encoded by *P. polymyxa* A18 genome

**Figure 4:**
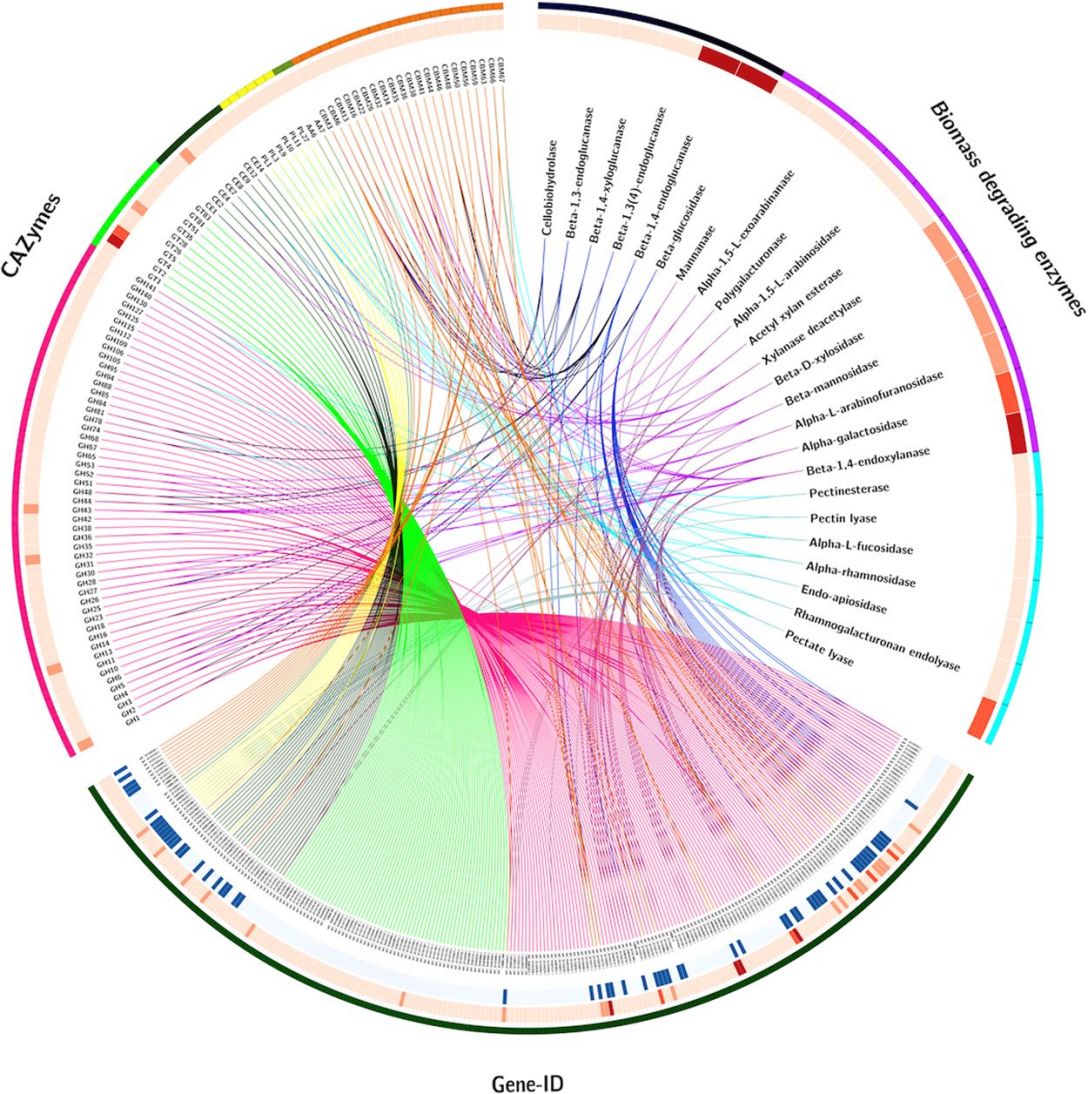
Carbohydrate-active enzyme (CAZymes) encoding genes present in *P. polymyxa* A18 genome including biomass degrading enzymes are represented. From outer to inner circle – Circle 1 - (**a**) Different CAZymes families GH (pink), GT (light green), CE (dark green), PL (yellow), AA (moss green) and associated module CBM (orange); (**b**) Classification on the basis of biomass degrading abilities-cellulose degrading enzymes (dark blue), hemicellulose degrading enzymes (purple) and pectin degrading enzymes (cyan); (**c**) Gene Ids of all the CAZyme genes in *P. polymyxa* A18. Circle 2 - Heatmap of different enzyme types (represented in light to dark red based on its count in the genome). Circle 3 - CAZymes having a signal peptide (represented in dark blue).

*P. polymyxa* A18 was selected in our previous study based on its ability to produce a high amount of cellulolytic enzymes, which hydrolyze plant biomass with high efficiency^6^. Therefore, we annotated the genome of *P. polymyxa* A18 for identifying genes encoding biomass hydrolyzing enzymes. The HMM profiles of CAZyme families present in DataBase for Carbohydrate-active enzyme ANnotation (dbCAN) (http://csbl.bmb.uga.edu/dbCAN/annotate.php) were used to annotate biomass hydrolyzing enzymes in the *P. polymyxa* A18 genome. Using this methodology, 280 domains were predicted across the genome of *P. polymyxa* A18 at the cutoffs for HMM alignment as mentioned in material and methods section. These domains were found to be encoded by 239 genes as some of the domains were present in duplicates or paired with other CAZy domains. Of the 239 genes encoding for carbohydrate-active enzymes, 234 were also annotated by NCBI as CAZymes, adding to their validation (Fig. 4; Additional file 2).

Of the 5 functional CAZy classes, the most abundant class found in *P. polymyxa* A18 genome was of glycoside hydrolases (GH); with a total number of 130 GH domains predicted in the genome (Fig. 4). Apart from GHs, 65 domains of glycosyl transferases (GT), 26 domains of carbohydrate esterases (CE), 12 of polysaccharide lyases (PL) and 2 of auxiliary activity (AA) enzymes were found (Fig. 4). A total of 45 carbohydrate binding modules (CBMs), which are generally associated with GHs for better substrate binding were also found in *P. polymyxa* A18. A detailed characterization of all CBMs of *P. polymyxa* A18, including their expression analysis and role in biomass hydrolysis, has been reported in our earlier study^28^. Categorization of CAZymes on the basis of functionality revealed that 76 CAZymes were predicted to be plant cell wall-degrading enzymes (CWDEs). Of these, 23 were cellulases, 35 hemicellulases and 18 pectinases (Supplementary Table S4). Amongst the cellulose hydrolyzing enzymes, β-1,4-endoglucanases (EGs, EC 3.2.1.4) of GH5 family and β-glucosidases (BGLs, EC 3.2.1.21) of GH1 and GH3 family were represented in the genome. A xyloglucan-specific endoglucanase of GH74 family associated with CBM3 and X2 was also encoded in the genome, as was reported before^28^. Hemicellulose, being a complex polymer, requires the action of several enzymes for its degradation^29^. Xylan, the major polysaccharide present in hemicellulose requires the cooperative action of endo-1,4-β-xylanase and xylan 1,4-β-xylosidase^29^. Xylan degrading enzymes like xylanase (3.2.1.8) of several GH families and β-D-xylosidase (3.2.1.37) of GH43 and GH52 were encoded by *P. polymyxa* A18 genome. Enzymes for galactomannan hydrolysis, i.e., β-mannanase, α-galactosidase, and β-mannosidase were present in abundance in the genome. Hydrolysis of hemicellulose is also facilitated by esterases. Acetyl xylan esterases liberate acetic acid esterifying position 2 and 3 on mono- and di-O-acetylated β-1,4-linked D-xylopyranosyl residues in xylan chains^30^. The genes encoding for these enzymes belonging to the CE1 family were present in *P. polymyxa* A18 genome. The endo-apiosidase belonging to GH140, which was shown recently to be involved in degradation of one of the most recalcitrant pectic polysaccharide of human diet by the gut bacteria^31^, was also found to be encoded by the *P. polymyxa* A18 genome.

Comparison of CAZymes amongst all the other members of *Paenibacillus* revealed some distinct variations in the numbers and families of these enzymes produced by different *Paenibacillus* species (Supplementary Fig. S1). These variations could emphasize the need of having different biomass hydrolysing properties to acclimatise to the natural habitats where *Paenibacillus* species live.

### Antibiotic resistance and synthesis in *P. polymyxa* A18

*P. polymyxa* A18 genome was analyzed for presence of protein coding sequences conferring resistance to antibiotics. A large number of such proteins were found to be encoded that provide resistance to a number of antibiotics, such as chloramphenicol, tetracycline, kanamycin, etc. Resistance to many antibiotics were verified experimentally (Table 1). Several multi-drug resistance transporters were also present in the genome, providing further selective advantage of *P. polymyxa* A18 in the insect gut environment.

**Table 1:** Genes responsible for conferring antibiotic resistance to *P. polymyxa* A18.

Antibiotics	Protein involved in conferring resistance	NCBI Protein ID	Experimental evidence (Minimum Inhibitory Concentration (MIC))
Chloramphenicol	Bcr/CflA subfamily of membrane proteins	WP_017427193.1	90 μg/ml
Daunorubicin	Daunorubicin resistance protein DrrA family ABC transporter ATP-binding protein	WP_017426002.1	ND
Erythromycin	Erythromycin esterase	WP_017426485.1	10 μg/ml
Fosfomycin	Fosfomycin resistance protein	WP_017428341.1	ND
Fosmidomycin	Fosmidomycin resistance protein	WP_017426391.1	ND
Kanamycin	Kanamycin nucleotidyl transferase	WP_017426690.1	120 μg/ml
Penicillin	BlaZ family class A beta-lactamase	WP_038978284.1	70 μg/ml
	penicillin-binding protein	WP_016820060.1	
	penicillin-binding protein 1F	WP_017427614.1	
Teicoplanin	Teicoplanin resistance protein VanZ	WP_017426835.1	ND
Tellurium	Tellurium resistance proteinTerA	WP_016821463.1	ND
Tetracycline	Tetracycline resistance MFS efflux pump	WP_016818770.1	90 μg/ml
		WP_017425800.1	
		WP_017426350.1	
		WP_017428077.1	
		WP_017428654.1	
Vancomycin	Vancomycin resistance protein	WP_038978182.1	20 μg/ml

ND - Not Done.

*P. polymyxa* strains are also known for their ability to produce a number of antibiotics^20^. These antibiotics are mostly peptides that are either ribosomally synthesized and post-translationally modified (RiPP) or non-ribosomally synthesized (NRPS). Analysis of *P. polymyxa* A18 genome using antiSMASH version 5.0^32^ indicated the presence of gene clusters coding for four antibiotics, i.e., paenibacillin (RiPP), polymyxin, tridecaptin and fusaricidin (NRPS) (Supplementary Table S5). A complete biosynthetic gene cluster encoding proteins for production, modification, regulation, immunity and transportation of paenibacillin, as reported by Huang and Yousef (2015)^33^, was detected in *P. polymyxa* A18 (Supplementary Table S5). Amongst the NRPS, a gene cluster coding for polymyxin comprising of genes *pmxA*, *pmxB* and *pmxE* for polymyxin synthetases and genes *pmxC* and *pmxD* for transport proteins^34^ were identified in the genome. Similarly, NRPS gene clusters for synthesis and transport of tridecaptin^35,36^ and fusaricidin^37,38^ were also predicted in *P. polymyxa* A18 genome, suggesting its ability to synthesize these antibiotics (Supplementary Table S5).

These peptide antibiotics are generally resistant to hydrolysis by peptidases and proteases because of their rigid and/or cyclic structures and presence of unusual constituents like D-amino acids^39^. Paenibacillin shows potency against Gram-positive bacteria^33^, while polymyxin and tridecaptin inhibit the growth of Gram-negative bacteria. Fusaricidin has been identified as a potential antifungal agent^37,38^. These wide spectra of antibiotics could give *P. polymyxa* a selective advantage for growth and colonization by preventing other microbes to compete for the host nutrition.

### Nitrogen metabolism

Members of the genus *Paenibacillus* can influence plant growth and health by furnishing nutrients to the plants by nitrogen fixation and are considered to be plant growth-promoting rhizobacteria (PGPR). Several species isolated from different kind of soils and plant rhizospheres have been found to be nitrogen-fixing strains amongst which *P. polymyxa* is one of the most studied^40^. In these rhizobacteria, nitrogen fixation is carried out by molybdenum-dependent nitrogenase encoded by *nif* gene cluster (*nifB*, *nifH*, *nifD*, *nifK*, *nifE*, *nifN*, *nifX*, *hesA*, and *nifV*)^10^. HGT, gene loss and duplication of these genes have contributed to the evolution of nitrogen fixation in *Paenibacillus*^41^. Inspection of the genome of gut isolate *P. polymyxa* A18 revealed that the *nif* gene cluster was absent in it (Supplementary Fig. S2). Analysis of this gene cluster in other members of *Paenibacillus* indicated that some of the related strains which are known to be PGPRs, like *P. polymyxa* CR-1, *P. polymyxa* SB3-1, and *P. terrae* HPL-003^10,42^, do have nitrogen-fixing genes (Supplementary Fig. S2). On the other hand genomes of *P. larva*, which is known to be an insect pathogen and resides in its gut, does not encode for *nif* gene cluster, suggesting this gene cluster is dispensable for the bacterial strains living in the gut.

Amongst the nitrogen metabolism genes, nitrate reductase-encoding genes *narGHJI* and nitrite reductase-encoding genes *nirBD* were present in the *P. polymyxa* A18 genome (Supplementary Fig. S2). These genes participate in dissimilatory nitrate reduction, i.e., they convert nitrate and nitrite to ammonia which can be excreted out. This feature might be helpful to the bacteria in removing excess nitrogen from the termite gut. These genes are present in almost all *P. polymyxa* and *P. larva* strains (Supplementary Fig. S2). The distribution of these genes suggests that members of the genus *Paenibacillus* acquire or retain genomic features based on the requirement of their ecological niches.

### Genome features associated with gut colonization in *P. polymyxa* A18

**Figure 5:**
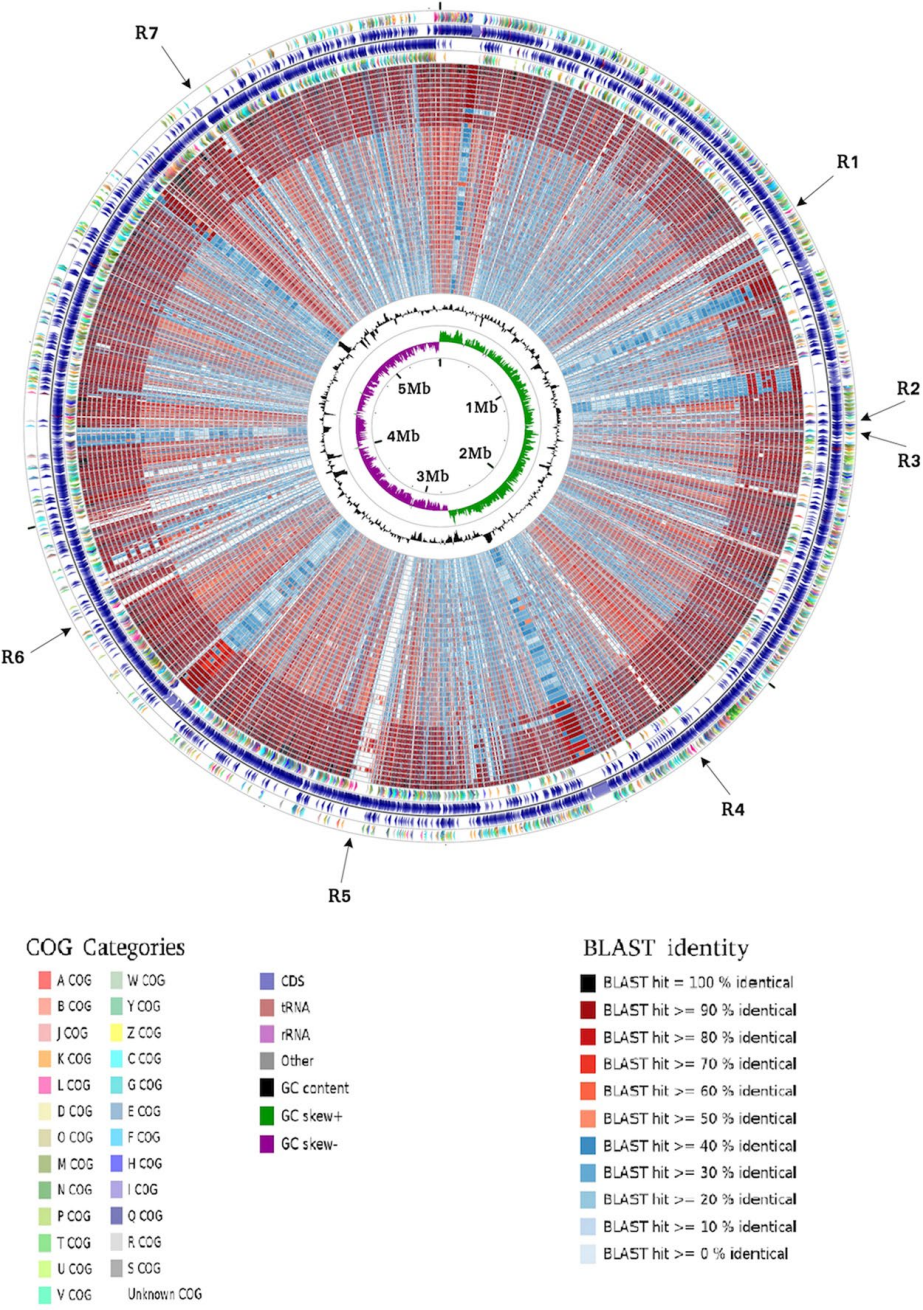
Circular representation of *P. polymyxa* A18 CDSs and their alignment with CDSs of members of the genus *Paenibacillus*. From outer to inner circle: the 1^st^ ring represents coding DNA sequences (CDS) of the plus strand of the *P. polymyxa* A18 chromosome under COG categories, the 2^nd^ ring represents total CDS of the plus strand, the 3^rd^ ring represents the total CDS of the minus strand, the 4^th^ ring represents CDS of the minus strand under COG categories, and the 5^th^ to 51^st^ rings represent identity matches of CDS of the strains with *P. polymyxa* A18 as mentioned in the order in Supplementary Table S3. The innermost rings represent the average GC content of the plus and minus strands and the GC-skew, respectively. Regions relevant to gut colonization have been represented as R1 to R7. Details have been mentioned in Table 2.

In order to further understand the ability of *P. polymyxa* A18 to reside in the gut, it was important to identify ORFs unique to *P. polymyxa* A18. To determine the unique regions, a BLAST-based comparative analysis was carried out. ORFs of *P. polymyxa* A18 were aligned to the ORFs of the 11 different strains of *P. polymyxa* and 36 other members of *Paenibacillus* genus (Fig. 5), whose genomes were completely sequenced and annotations were available at NCBI database.

Circular map of the ORF alignment displayed variations in homology of ORFs across members of *Paenibacillus* (Fig. 5). Some of the regions in the ORF map showed high homology to ORFs present in most of the *P. polymyxa* species but low or no homology with other *Paenibacillu*s species (denoted as regions R2, R3, and R7 in Fig. 5 with zoom map in supplementary Fig. S3). These regions were found to have ORFs that encode for machinery required for assembly of adhesin proteins (R2), Type IV pili (R3) and flagella (R7) (Table 2). All of these features play important role in biofilm formation, colonization and pathogenesis in a number of genera of bacteria. The complete set of *tad* (tight adherence) genes required for assembly of fimbrial low-molecular-weight pili were present on a genomic island named the widespread colonization island (WCI) in region R3. The WCI was also identified in two other *Paenibacillus* strains, *P. macerans* and *Paenibacillus sp*. strain JCM10914, which were earlier isolated from the termite gut^43,44^. This provides an indication that these proteins could aid in adherence to the gut wall and hence survival in the gut.

**Table 2:** Localization of gut-specific features in the genome of *P. polymyxa* A18.

Region No.	Name of Loci	Genomic Region	NCBI Accession no.	Product description
R1.	Type I Restriction Modification System	897034..898581	WP_038978098.1	Restriction endonuclease, DNA-methyltransferase subunit M
		898578..899840	WP_017426209.1	Type I RM system, specificity subunit S
		901071..904169	WP_017426212.1	Type I RM system, restriction subunit R
		904193..904486	WP_017426213.1	Type I RM system, DNA-methyltransferase subunit M
R2.	Adhesin	1409160..1409930	WP_017426498.1	Large adhesin
R3.	Widespread Colonization Island (including Type IV pilli)	1481503..1482750	WP_017426545.1	TadA
		1482747..1483538	WP_017426546.1	TadB
		1483551..1484429	WP_038978149.1	TadC
		1484440..1484634	WP_016820682.1	Hypothetical Protein
		1484634..1485302	WP_017426549.1	TadE
		1485343..1487559	WP_017426550.1	Hypothetical Protein
		1487528..1488523	WP_017426551.1	Hypothetical Protein
		1488611..1489123	WP_026065394.1	TadV
		1489185..1490969	WP_017426553.1	Forkhead Associated Protein
R4.	CRISPR/Cas System	2210905..2213388	WP_017427970.1	Cas3
		2213502..2214218	WP_017427971.1	Cas5d
		2214219..2216126	WP_017427972.1	Csd1
		2216209..2217084	WP_017427973.1	Cas7
		2217071..2217730	WP_017427974.1	Cas4
		2217727..2218758	WP_017427975.1	Cas1
		2218769..2219059	WP_017427976.1	Cas2
R5.	Phage related proteins	complement (2996310.. 3036881)	—	Phage proteins
		3037704..3038891	WP_016822090.1	Phage integrase
R6.	CRISPR/Cmr System	complement (3761099..3761932)	WP_017428210.1	Cmr6
		complement (3761952..3762323)	WP_017428211.1	Cmr5
		complement (3762320..3763279)	WP_017428212.1	Cmr4
		complement (3763269..3764558)	WP_049885704.1	Cmr3
		complement (3764527..3766347)	WP_038978046.1	Cmr2
		Complement (3766347..3767900)	WP_017428215.1	Cmr1
R7.	Flagellar Biosynthesis Proteins	Complement (5061524..5063260)	WP_038978178.1	Motility accessory factor
		Complement (5063812..5064195)	WP_017427105.1	Flagellar biosynthesis protein FliS
		Complement (5064210..5065682)	WP_017427106.1	Flagellar hook-associated protein FliD
		Complement (5065696..5066073)	WP_017427107.1	Flagellin protein FlaG
		Complement (5066242..5067015)	WP_017427108.1	Flagellin protein FlaA
		Complement (5067559..5068005)	WP_017427110.1	Flagellar assembly factor FliW
		Complement (5068089..5068997)	WP_017427111.1	Flagellar hook-associated protein FlgL
		Complement (5069022..5070602)	WP_017427112.1	Flagellar hook-associated protein FlgK

Certain unique regions were detected across the genome of *P. polymyxa* A18 which were poorly represented in the genomes of other *P. polymyxa* strains. These regions might encode certain features that could be associated with its ability to sustain the gut environment. Importantly, the location of these regions in the genome coincided with the regions acquired by A18 via horizontal gene transfer, as indicated by the GC content of these regions which deviated from the average GC content of *P. polymxa* A18 genome (regions R1, R4, and R6 in Fig. 5). These regions encoded for Type I Restriction-Modification (RM) system (R1) and CRISPR/Cas system (R4 and R6) (Table 2). RM and CRISPR/Cas system confers resistance to the host bacteria against foreign genetic elements such as bacteriophages and plasmids. A recent report suggests that RM system along with CRISPR element act synergistically in defense against bacteriophages^19^. Insect gut is known to be a reservoir of various types of viruses^45^. The features present in *P. polymxa* A18 indicated that these mechanisms might be serving to combat the high viral load in the gut of insects. CRISPR/Cas system abrogates the replication of viruses or plasmids^46^ by encoding proteins with nucleic acid-manipulative activities such as nucleases, helicases, and polymerases^47,48^. Three types of CRISPR/Cas systems have been defined so far^48^. The Type I and Type II systems target double-stranded DNA while the Type III system target single-stranded RNA. Two stretches of DNA, one encoding series of Cas proteins arranged in the fashion cas3-cas5-cas8-cas7-cas4-cas1-cas2 and second encoding series of Cmr (CRISPR RAMP Module) proteins arranged in the fashion cmr1-cmr2-cmr3-cmr4-cmr5-cmr6 were found in the *P. polymyxa* A18 genome (Table 2, Supplementary Fig. S4). These arrangements suggested that *P. polymyxa* A18 had both Type I-C and Type III-B CRISPR/Cas systems, respectively^49^. The Type I-C system has been reported in *Bacillus halodurans* (alkalophilic bacterium) and *Mannheimiasuccinici producens* (bovine rumen bacterium)^50,51^ whereas Type III-B system has been reported in *Pyrococcus furiosus, Thermus thermophilus and Sulfolobus solfataricus*^52–54^. Since Type I-C and Type III-B systems are prevalent in extremophiles, there is a possibility of lateral gene transfer from these bacteria in the gut environment.

### *In situ* host colonization

All the unique genomic loci identified in *P. polymyxa* A18 (mentioned in Table 2) pointed towards its ability to colonize the gut environment. To further demonstrate that *P. polymyxa* A18 has the ability to colonize the termite gut and it wasn’t present there accidentally when the gut dissection was done, the survival rate of *P. polymyxa* A18 inside the gut was monitored over a defined period of time. *Paenibacillus sp*. JDR-2, which is a free-living bacterium and its genome does not code for specific features associated with gut colonization (Fig. 5, Supplementary Fig. S4 and Table 2), served as a control in our experiment for *P. polymyxa* A18 colonization.

**Figure 6:**
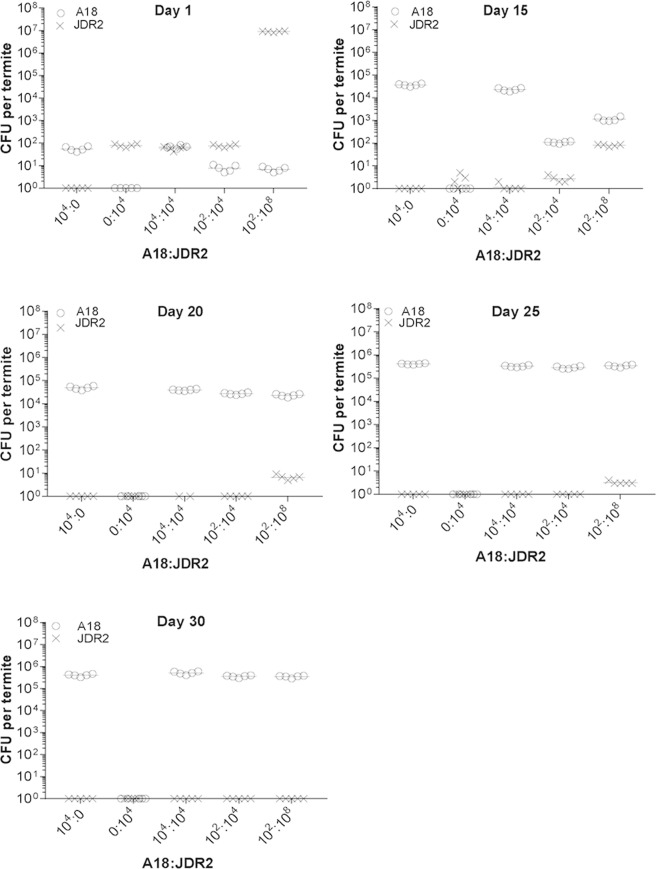
Host specificity of *P. polymyxa* A18 as shown by colonization efficiency in termite. Gut bacterial load (CFU) was counted from day 1 after oral inoculation. The sample size of termite for each treatment was 5. Means of bacterial counts from 5 termites are shown as a straight line. The ratios of *P. polymyxa* A18 and *Paenibacillus* JDR-2 (represented as A18: JDR2) used in various experiments are shown in the X-axis.

Termite guts were first sterilized by adding antibiotic (rifampicin) into their feed and then fed with cotton containing onetime inoculum of 10^4^ cfu of either *P. polymyxa* A18 or *Paenibacillus sp*. JDR-2 (represented as 10^4^:0 and 0:10^4^ for A18: JDR2 in Fig. 6 to show the inoculation either for A18 or JDR2, respectively). Their colonization success was monitored for over 30 days as follows. At regular intervals termite guts were dissected, plated on antibiotic selection plates and cfu (colony forming units) were counted. The cfu count for both *P. polymyxa* A18 and *Paenibacillus sp*. JDR-2 showed a value of ~10^2^ on day 1 (Fig. 6). While cfu count increased to 10^5^ on day 15 for *P. polymyxa* A18 and further higher on day 25 samples, the colony count of *Paenibacillus sp*. JDR-2 declined simultaneously (Fig. 6). *P. polymyxa* A18 and *Paenibacillus sp*. JDR-2 were also co-inoculated in equivalent amounts of 10^4^:10^4^ cfu and it was found that only *P. polymyxa* A18 could multiply and increase in numbers in the gut. To test if *P. polymyxa* A18 can colonize despite a numerical disadvantage, *P. polymyxa* A18 and *Paenibacillus sp*. JDR-2 were coinoculated in the ratios 10^2^:10^4^ and 10^2^:10^8^ cfu. All coinoculated samples reached cfu levels comparable to mono-inoculated samples, and almost all recovered colony-forming units were of *P. polymyxa* A18 (Fig. 6). While *P. polymyxa* A18 appeared on plates at all days from the gut sample after feeding, *Paenibacillus sp*. JDR-2 was not observed on plates after feeding *Paenibacillus sp*. JDR-2. Thus, gut bacteria, similar to *P. polymyxa* A18, have the ability to overcome a large numerical disadvantage and completely outcompete free-living bacteria to colonize the gut.

## Discussion

Microbial plant cell wall degraders play a pivotal role in recycling of photosynthetically fixed carbon. Only a small fraction of microorganisms are specialized to hydrolyze the recalcitrant structure of cellulose present in plant cell walls. One such microorganism, *P. polymyxa* A18, was identified from the termite gut to possess high cellulolytic and hemicellulolytic activities^6^. We sequenced, assembled and analyzed the genome of *P. polymyxa* A18 to get insights into features that enable it to be an efficient termite symbiont. The total assembled genome size and the number of genes identified in its genome were similar to that of other *P. polymyxa* genomes, though they varied significantly among different species of *Penibacillus* (Fig. 3, Supplementary Table S3). A large inventory of cell wall degrading CAZymes was annotated in its genome, which was classified as CWDEs (Fig. 4). Some variations in numbers of CAZymes encoded by the genome were noted amongst different *Paenibacillus* species, particularly in *Paenibacillus sp*. JDR-2, *P. mucilaginous* KNP414, and *P. terrae* HPL-003, which follow different lifestyles (Supplementary Fig. S1). Large numbers of the glycoside hydrolases encoded in the genome were found to have specificities towards amorphous and short chain cellulose (Fig. 4). One-sixth of the glycoside hydrolases are associated with carbohydrate binding modules indicating the ability of these enzymes to bind to crystalline portions of cellulose. Functional characterization of some of these enzymes of *P. polymyxa* A18 has been reported before from our laboratory^28^.

Genome analysis further enabled us to identify mobile elements like insertion sequences and prophages in the genome. Two major sites were located in the genome with phage DNA integrations (Fig. 5), indicating high viral load in the surroundings of this gut isolate. Amongst the genes transferred to the genome of *P. polymyxa* A18 through horizontal gene transfers, two major loci having a role in foreign DNA modification, i.e., Restriction-Modification System-I and CRISPR/Cas system, were noted (Table 2). Presence of these along with other genetic elements, such as those which codes for machinery required for assembly of adhesin proteins, Type IV pili and flagella, indicated that *P. polymyxa* A18 might have adapted itself to survive in the gut as against the previously characterized members of *Penibacillus* which were known to be free-living and plant growth promoting.

To further validate this argument, other available sequenced genomes of the members of genus *Paenibacillus* were compared. Genome sequence alignment and its analysis suggested that CRISPR/Cas and RM-I system were unique to the gut isolate *P. polymyxa* A18 genome. These properties not only indicated a high viral load in the gut environment but also showed the development of machinery to overcome the gut viral load. Also, the presence of gene families that code for proteins like adhesins and Type IV pili were shown to be present in other gut symbionts, such as those of honey bees *Apis mellifera* and bumble bees *Bombus impatiens*^18^, aiding to their colonization in the gut. The colonization ability of *P. polymyxa* A18 was further established experimentally in termite, where it outcompeted the non-colonizing bacterium *Paenibacillus* sp JDR-2. While *P. polymyxa* A18 seemed to have retained some of the features that may have helped it further to exist in the gut, such as those involved in nitrate and nitrite metabolism, antibiotic resistance and synthesis, etc, it has disposed some of them which are not necessary in the gut environment, such as those involved in nitrogen fixation (Supplementary Fig. S2).

Overall, our findings indicate that the *P. polymyxa* A18 may have acquired or retained genome features to establish itself in the gut of termite and, at the same time, it provides assistance to termites to digest their food in a symbiotic relationship by secreting wide varieties of lignocellulolytic enzymes. The information obtained from this study will help not only in the identification of important enzymes for biomass hydrolysis but also contribute to the understanding of genome features specific to the specific niches of the organisms.

## Methods

### Genome sequencing of *P. polymyxa* A18

*Paenibacillus polymyxa* A18 was cultivated in tryptone soy broth (TSB, Himedia) and its genomic DNA was isolated using genomic DNA Isolation Kit (Qiagen) according to the manufacturer’s protocol. An FLX shotgun library and an 8-kb paired-end library were prepared for sequencing using the GS-FLX Titanium platform (Roche454, Branford, USA). Quality-filtered sequences from whole genome shotgun sequencing were assembled using the GS De Novo Assembler (http://454.com/products-solutions/analysis-tools/gs-de-novo-assembler.asp). Reads that overlapped each other were joined into contigs, which were further joined to form scaffolds using paired-end sequencing data. This whole genome shotgun project was deposited at DDBJ/EMBL/GenBank under accession number JWJJ00000000. The version described in this study is JWJJ01000000. *P. polymyxa* A18 genome sequence was circularized using *P. polymyxa* M1 genome as a template in CONTIGuator^55^. Further genome analysis was performed using circularized genome.

### Gene prediction and annotation of *P. polymyxa* A18 genome

For gene prediction and annotation, the genome assembly was submitted to the Rapid Annotations using Subsystems Technology (RAST) Server (http://rast.nmpdr.org/)^22^ and National Centre for Biotechnology Information (NCBI) prokaryotic annotation pipeline. Annotations obtained from both the pipelines were compared and only the genes predicted by both the methods were used for further analysis. Genome features such as insertion sequences were predicted using IS Finder database at a cutoff of 0.5^56^. Tandem repeats were identified using tandem repeat finder program. Phage integrations were predicted using PHAST^57^. Horizontal gene transfers were identified using Island viewer. For identification of tRNA encoding regions in the genome tRNA scan-SE 1.21^58^ was used. Proteins were characterized as secretory by determining signal peptides using SignalP^59^. Transmembrane domains were predicted using TMHMM Server v2.0 and only protein domains containing a minimum of 20 amino acids in the transmembrane region were annotated as transmembrane proteins. Artemis was used for visualization of the genome and the gene annotations^60^.

### Prediction of Carbohydrate-Active Enzyme (CAZy) in members of genus *Paenibacillus*

CAZy domains were identified in *P. polymyxa* A18 and other members of genus *Paenibacillus* using HMMER hmmscan. HMM profiles of CAZy families were downloaded from dbCAN (release 6.0)^61^ and the hmmscan program in HMMER 3.0 package^62^ was used to search for these domains in CDS encoded in the genome. Primary results were processed by the parser script supplied by the dbCAN. E value cut-offs for CAZy categories were optimized on the basis of results matching with the CAZy database (http://www.cazy.org/)^63^ (AA 10^−11^, CBM 10^−12^, CE 10^−22^, GH 10^−16^, GT 10^−5^ and PL 10^−11^). Circos tool^64^ was used to visualize the links between the genes, CAZy family domain and its function.

### Antibiotic susceptibility testing

*P. polymyxa* A18 was tested for susceptibility to different concentrations of the antibiotics using the Kirby–Bauer disc diffusion method. The following antibiotics obtained in powdered form from Sigma-Aldrich (St. Louis, MO) were used: Tetracycline (T7660), Erythromycin (E6376), Kanamycin (K4000), Chloramphenicol (C0378) Penicillin (13752) and Vancomycin (V2002). Stock solutions (2.0 mg/ml) of the antibiotics were prepared in the appropriate diluents and filter sterilized using 0.22-μm syringe filter. The aliquots (1 ml) of the stock solutions were stored at −20 °C until use (not exceeding a 4-week storage period). Disks containing ranges of antibiotic concentrations - Tetracycline (1–150 µg/ml), Erythromycin (1–25 µg/ml), Kanamycin (10–150 µg/ml), Chloramphenicol (10–150 µg/ml), Penicillin (10–100 µg/ml) and Vancomycin (10–90 µg/ml), were placed on the lawn of *P. polymyxa* A18 cultivated on Tryptone soy agar plate (individual concentration of each antibiotic used is available in Supplementary Table S6). Plates were incubated overnight at 37 °C. The Minimum Inhibitory Concentration (MIC) was defined as the lowest concentration of antibiotic which gives a complete inhibition of visible growth in vicinity of the disk. All MIC experiments were performed in triplicate on the same day.

### Phylogenetic analysis

The pan-genome analysis was done to identify core genes using the Bacterial Pan Genome Analysis (BPGA) tool^65^. The amino acid sequences of each core genes were aligned individually using the MAFFT (Multiple sequence Alignment based on Fast Fourier Transform) program^66^, then each alignment of core genes was concatenated. The concatenated core gene alignment was used for computing the phylogenetic tree based on a maximum likelihood algorithm with a bootstrap value of 1000 and PROTCATAUTO parameter using the RAxML-HPC^67^. The phylogenetic tree was visualized using FigTree (1.4.3)^68^.

### Comparative genomics

For comparison of *P. polymyxa* A18 genome with other strains belonging to *Paenibacillus* genus, only those members whose complete whole genome sequences were available at NCBI were used for analysis. The accession numbers of strains used here for the analysis are provided in Supplementary Table S3. For genome comparison, each of the above-mentioned members of the genus *Paenibacillus* was aligned to the reference genome *P. polymyxa* A18 using BLAST Ring Image Generator (BRIG)^69^. CG View Comparison Tool (CCT) was used to generate circular CDS comparison map of *P. polymyxa* A18 with other members of the genus *Paenibacillus*^70^.

### Gut colonization experiments

Experiments to establish colonizing capabilities of *P. polymyxa* A18 and *Paenibacillus sp*. JDR-2 in termites were conducted as follows. Termites were kept in a 25 °C incubation chamber under moist conditions and fed with wet cotton for 24 hr. Termite gut was then sterilized by feeding cotton saturated with 100 µg/ml rifampicin solution for the next 24 hr. After feeding the antibiotic, two termites from each set were dissected and confirmed for sterility by plating the homogenate on TSB agar plate. Once the sterility was confirmed, termites were fed with water for 24 hr to remove traces of antibiotic from the gut. Sterility was again ensured at this stage by dissecting two termites. Termites were then fed with *P. polymyxa* A18 and *Paenibacillus sp*. JDR-2 for 24 hr and then allowed to feed on water. The guts of treated termites were isolated at regular intervals from 1^st^ day onwards up to the 30^th^ day. Every day, dead individuals were removed and Petri dishes and filter papers were replaced to avoid re-inoculation through feces. At all stages, a control set was maintained wherein termites were fed with wet cotton only. Isolated guts were allowed to grow on TSA plates and individual colonies were counted. To confirm gut colonization of *P. polymyxa* A18 and *Paenibacillus sp*. JDR-2, colonies were counted on antibiotic selection plates. 60 µg/ml vancomycin was used for selection of JDR-2 and 50 µg/ml kanamycin was used for selecting A18 as they were found resistant towards these antibiotics.

## Supplementary information

Supplementary Figures and Tables

Additional file 1

Additional file 2

## Data Availability

All data generated or analysed during this study are included in this published article (and its Supplementary Information files).
