# Supplementary material for: Genome analysis of Paenibacillus polymyxa A18 gives insights into the features associated with its adaptation to the termite gut environment
Source: Sci Rep. 2019 Apr 15;9:6091. doi: 10.1038/s41598-019-42572-5 (PMC6465253; doi:10.1038/s41598-019-42572-5)
Supplement: Supplementary file 1 — Supplementary Figures and Tables [file 41598_2019_42572_MOESM1_ESM.pdf]

Supplementary Information

Genome analysis of *Paenibacillus polymyxa* A18 gives insights into the features associated with its adaptation to the termite gut environment

Nandita Pasari[1,2], Mayank Gupta[1,2], Danish Eqbal[1], Syed Shams Yazdani[1,2,*]

Supplementary information provided with this submission:

Figure S1. Comparative analyses of Carbohydrate-active enzyme (CAZymes) numbers across the members of the genus *Paenibacillus.*

Figure S2. Comparative analyses of genes involved in nitrogen fixation and dissimilatory nitrate reduction across the members of the genus *Paenibacillus.*

Figure S3. Zoom map of circular representation of P. polymyxa A18 CDSs and their alignment with CDSs of members of the genus Paenibacillus for regions R2, R3 and R7 as mentioned in Figure 4.

Figure S4. Comparative analyses of genes encoding for CRISPR/Cas and CRISPR/cmr across the members of the genus *Paenibacillus.* The colored box represents the presence of a gene within the genome and white box indicates the absence of a gene.

Table S1: General features of *P. polymyxa* A18 genome

Table S2: Horizontal Gene Transfers in *P. polymyxa* A18 genome

Table S3: Genome features of members belonging to genus *Paenibacillus*

Table S4: Categorization of CAZy genes encoded in *P. polymyxa* A18 according to their functions

Table S5: Genes encoding for antibiotic synthesizing polypeptides in *P. polymyxa* A18

Table S6. Concentration of antibiotics used for susceptibility testing

Additional file1: Annotation of *P. polymyxa* A18 ORFs

Additional file2: Annotation of CAZy genes in *P. polymyxa* A18

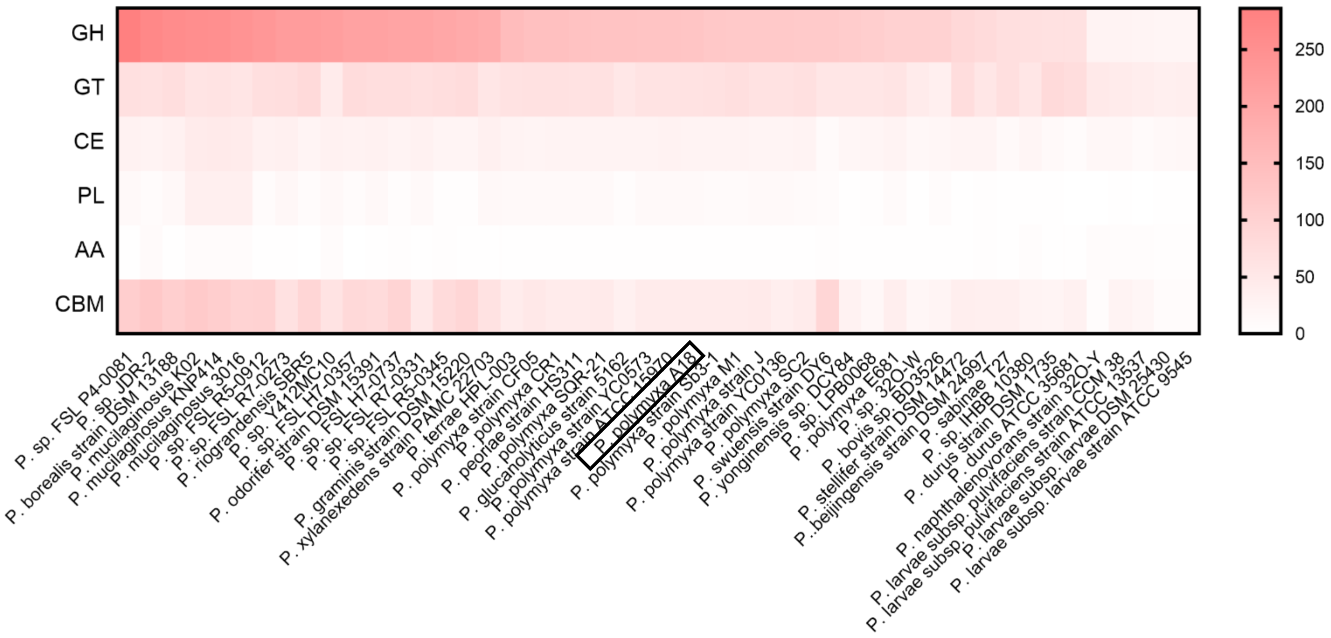

Figure 1. Comparative analyses of Carbohydrate-active enzyme (CAZymes) numbers across the members of the genus *Paenibacillus*. *P. polymyxa* A18 has been represented in a black outlined box. Abbreviations - GH, glycoside hydrolase; GT, glycosyl transferase; CE, carbohydrate esterase; PL, polysaccharide lyase; AA, auxiliary activity; CBM, carbohydrate-binding module.

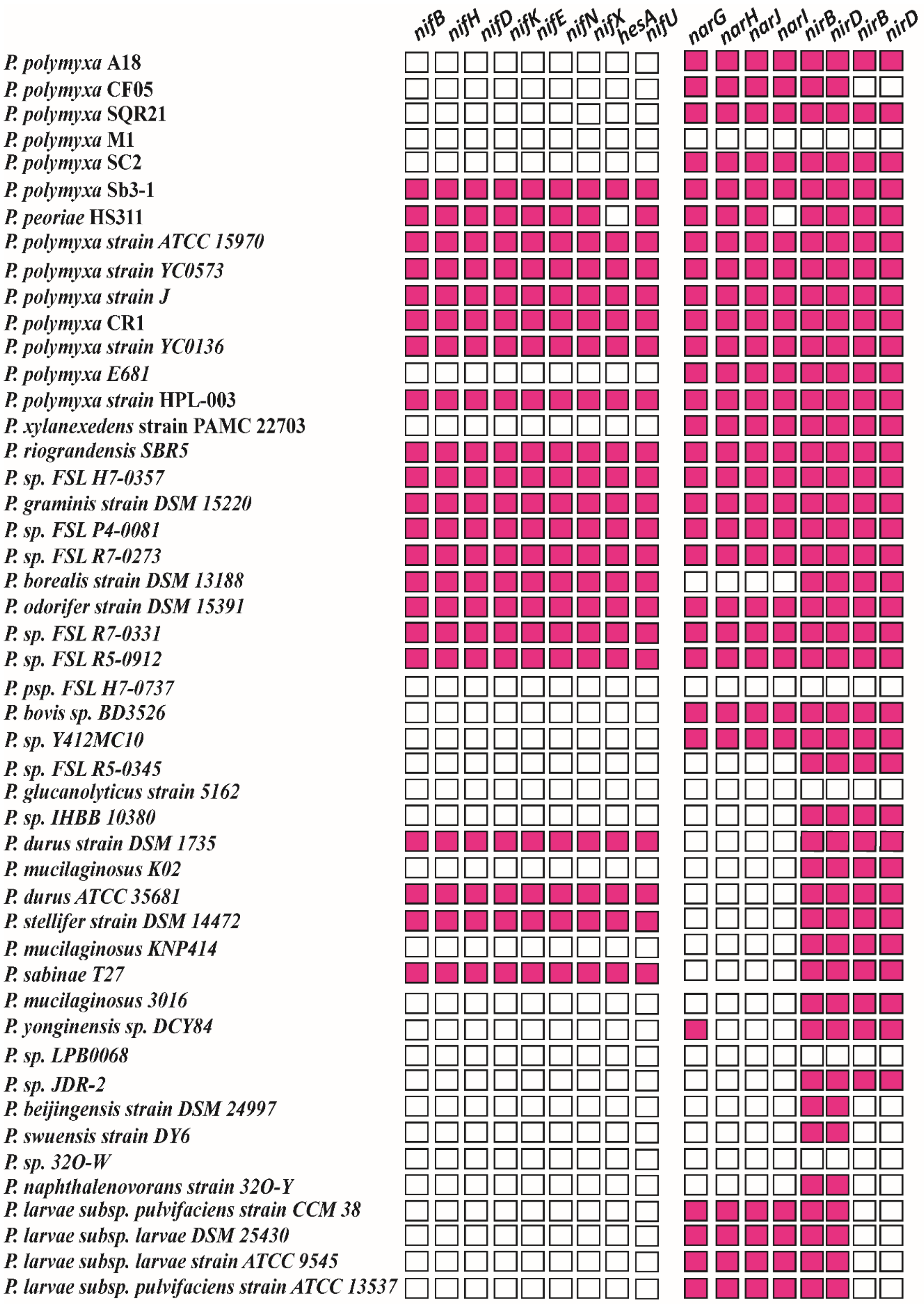

Figure S2. Comparative analyses of genes involved in nitrogen fixation and dissimilatory nitrate reduction across the members of the genus *Paenibacillus*. The colored box represents the presence of a gene within a genome and white box indicates the absence of a gene.

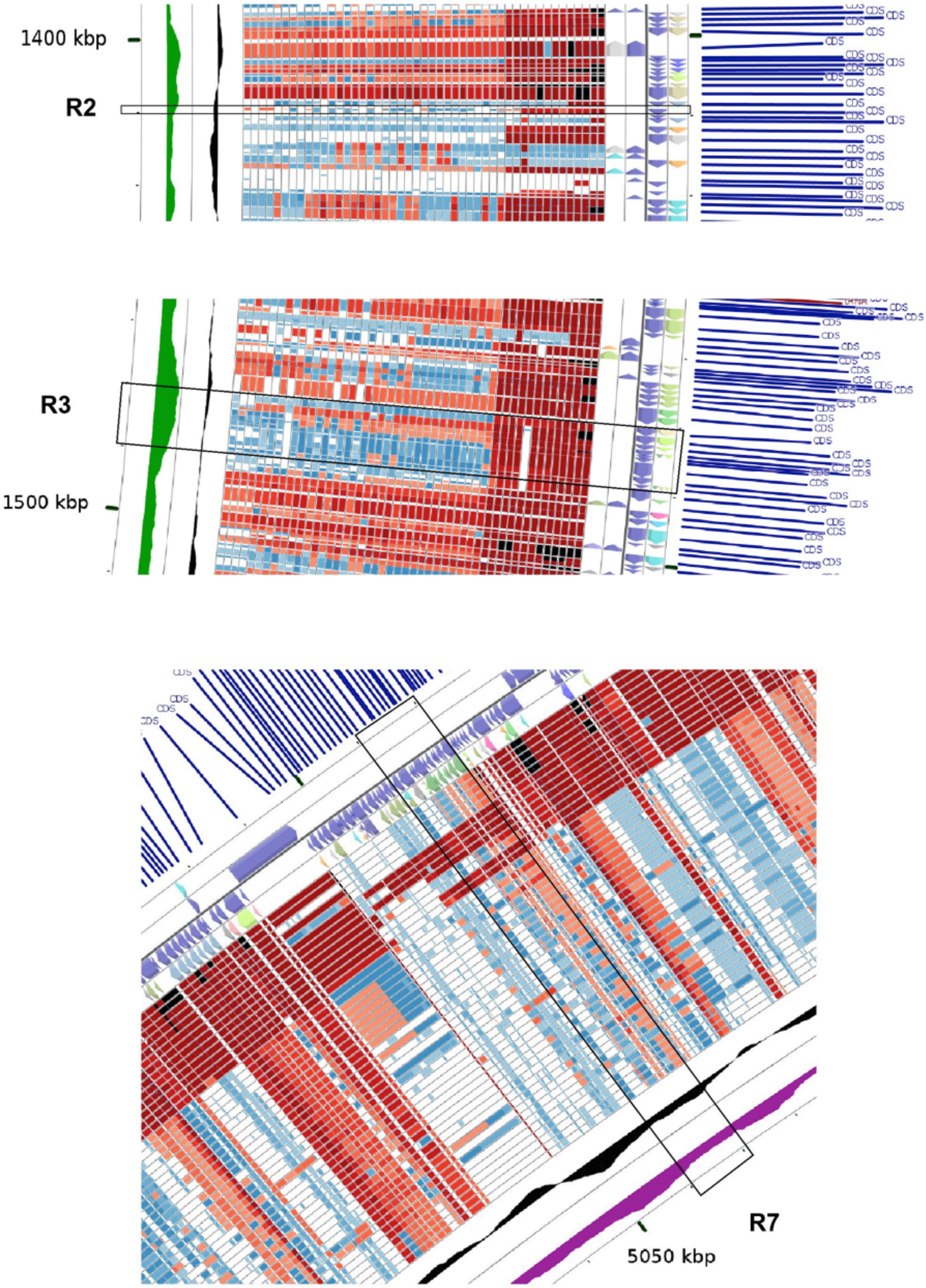

Figure S3. Zoom map of circular representation of *P. polymyxa* A18 CDSs and their alignment with CDSs of members of the genus *Paenibacillus* for regions R2, R3 and R7 as mentioned in Figure 4.

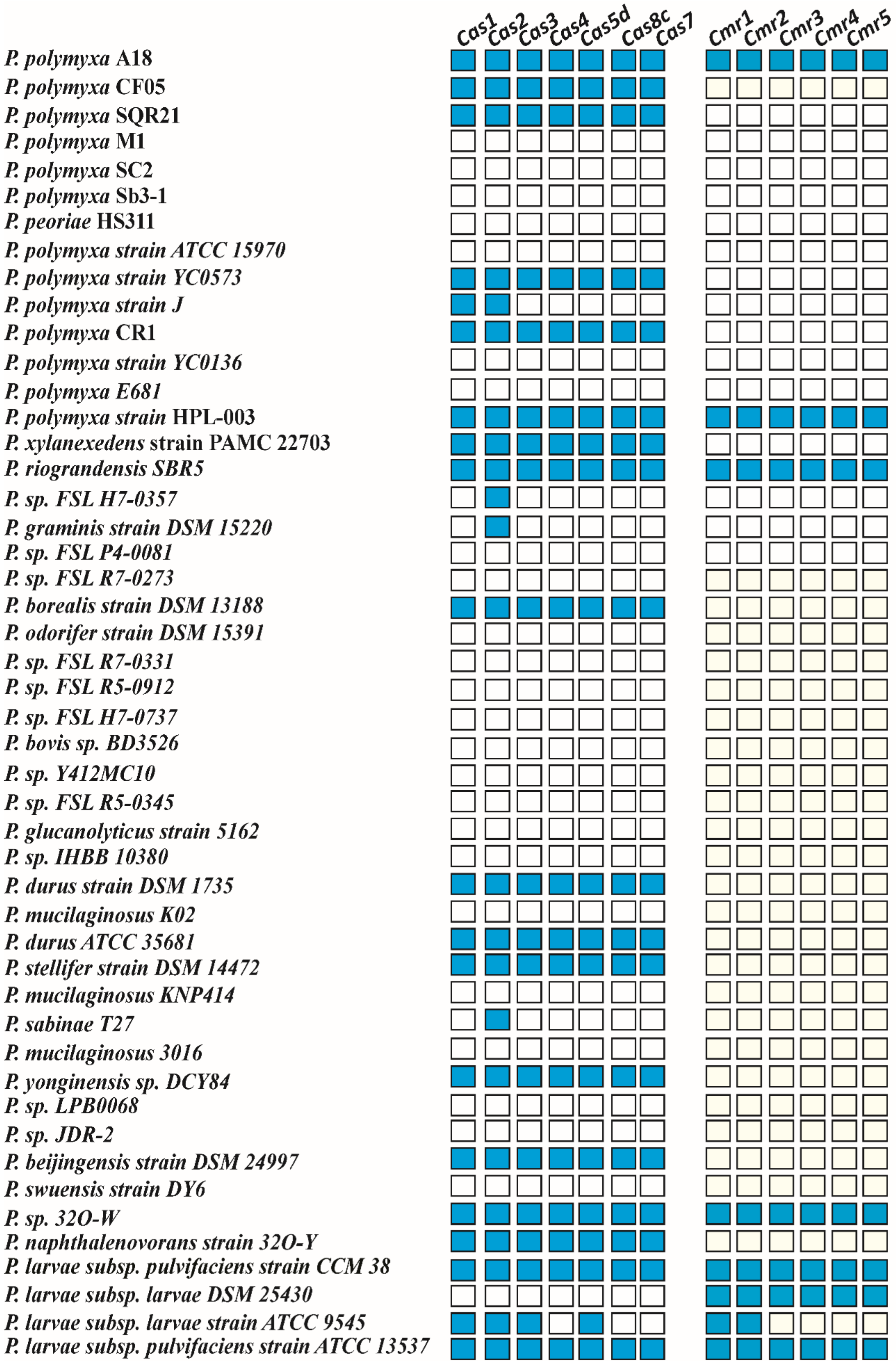

Figure S4. Comparative analyses of genes encoding for CRISPR/Cas and CRISPR/cmr across the members of the genus *Paenibacillus.* The colored box represents the presence of a gene within the genome and white box indicates the absence of a gene.

Table S1: General features of *P. polymyxa* A18 genome

Sequencing platform	Roche 454
Genome size	5.72 Mb
Number of contigs	682
Number of scaffolds	28
Sequencing coverage	16.5x
GC content (%)	46.2
Predicted CDS	4608
tRNAs	105
Mobile element genes/ transpoases	23

Table S2: Horizontal Gene Transfers in *P. polymyxa* A18 genome

Island start	Island end	Length	Gene start	Gene end	Product
333544	340568	7024	333544	334965	arylsulfatase regulator
			334955	335833	ABC transporter ATP-binding protein
			335838	336527	hypothetical protein
			336524	337669	hypothetical protein
			337736	339583	Lipid A export ATP-binding/permease protein MsbA
			340362	340568	hypothetical protein
512341	536062	23721	512502	513248	3-oxoacyl-[acyl-carrier protein] reductase (EC 1.1.1.100)
			513741	514949	Mobile element protein
			515161	515322	hypothetical protein
			515555	515980	hypothetical protein
			516572	516754	hypothetical protein
			516825	517922	Spore germination protein GerKB
			517951	519177	Spore germination protein GerKC
			519190	520806	Spore germination protein GerKA
			521056	521184	hypothetical protein
			522225	522620	unknown
			523774	524685	Transcriptional regulator, AraC family
			524799	525092	hypothetical protein
			525043	525609	putative short-chain dehydrogenase/oxidoreductase
			525820	526647	AP endonuclease, family 2
			526664	528010	Fructoselysine transporter FrlA, cationic amino acid permease
			528171	529184	Glucosamine-fructose-6-phosphate aminotransferase [isomerizing] (EC 2.6.1.16)
			529279	530061	Fructoselysine kinase (EC 2.7.1.-)
			530125	530853	Transcriptional regulator of fructoselysine utilization operon FrlR
			531125	531775	hypothetical protein
			532132	533115	kelch-like 1
			533325	533558	hypothetical protein
			533687	534502	hypothetical protein

			534705	535247	Ribosomal protein S5p alanine acetyltransferase
			535718	536062	Dienelactone hydrolase family
897034	901038	4004	897034	898581	Type I restriction-modification system, DNA-methyltransferase subunit M (EC 2.1.1.72)
			898578	899840	Type I restriction-modification system, specificity subunit S (EC 3.1.21.3)
			899842	901038	Anticodon nuclease
1730296	1738714	8418	1730296	1731372	6 kDa early secretory antigenic target ESAT-6 (EsxA)
			1731384	1731605	hypothetical protein
			1732278	1732736	hypothetical protein
			1732912	1733160	hypothetical protein
			1733814	1733978	hypothetical protein
			1734127	1734399	hypothetical protein
			1734552	1734719	hypothetical protein
			1734732	1734974	hypothetical protein
			1734976	1735089	hypothetical protein
			1735211	1735498	hypothetical protein
			1735884	1736429	hypothetical protein
			1737097	1738305	Mobile element protein
1731384	1736429	5045	1731384	1731605	hypothetical protein
			1732278	1732736	hypothetical protein
			1732912	1733160	hypothetical protein
			1733814	1733978	hypothetical protein
			1734127	1734399	hypothetical protein
			1734552	1734719	hypothetical protein
			1734732	1734974	hypothetical protein
			1734976	1735089	hypothetical protein
			1735211	1735498	hypothetical protein
			1735884	1736429	hypothetical protein
2115218	2121318	6100	2115218	2115742	hypothetical protein
			2115739	2116386	hypothetical protein
			2116794	2117135	Drug resistance transporter EmrB/QacA subfamily
			2117506	2117625	hypothetical protein
			2117581	2118279	SSU ribosomal protein S2p (SAe)
			2118412	2119062	Translation elongation factor Ts
			2119183	2119911	Uridine monophosphate kinase (EC 2.7.4.22)
			2119911	2120465	Ribosome recycling factor
			2120551	2121318	Undecaprenyl diphosphate synthase (EC 2.5.1.31)
2334173	2341097	6924	2334173	2334295	D-alanyl-D-alanine carboxypeptidase (EC 3.4.16.4)
			2334438	2335025	Peptidoglycan N-acetylglucosamine deacetylase (EC 3.5.1.-)
			2334998	2335174	hypothetical protein
			2335167	2335718	hypothetical protein
			2335735	2336304	putative integral membrane protein
			2336329	2337495	1,2-diacylglycerol 3-glucosyltransferase (EC 2.4.1.157)
			2337526	2338227	Peptidoglycan N-acetylglucosamine deacetylase (EC 3.5.1.-)
			2338250	2339383	diglucosyldiacylglycerol synthase

			2339380	2340606	Multidrug-efflux transporter
			2340789	2341097	Alkaline phosphatase like protein
2419508	2461861	42353	2419508	2461861	Bacitracin synthetase 3 (BA3)
2669891	2682305	12414	2669891	2670901	Two-component response regulator yesN
			2671332	2672312	Beta-galactosidase (EC 3.2.1.23)
			2672328	2673701	PTS system, beta-glucoside-specific IIB component (EC 2.7.1.69) / PTS system, beta-glucoside-specifi
			2673736	2676159	Beta-glucosidase (EC 3.2.1.21)
			2676291	2677472	Beta-lactamase class C
			2677530	2678399	Gluconolactonase (EC 3.1.1.17)
			2679006	2681672	Possible DNA-binding protein
			2681760	2682305	PTS system, beta-glucoside-specific IIB component (EC 2.7.1.69)
3026847	3038959	12112	3026920	3027102	hypothetical protein
			3027099	3027287	hypothetical protein
			3027410	3027598	hypothetical protein
			3027722	3027940	hypothetical protein
			3027965	3028192	hypothetical protein
			3028189	3028503	hypothetical protein
			3028454	3028972	Hypothetical protein, Lmo2306 homolog [Bacteriophage A118]
			3028973	3030091	DNA polymerase III beta subunit (EC 2.7.7.7)
			3030220	3031176	Helicase loader DnaI
			3031183	3032193	Phage replication initiation
			3032228	3032650	hypothetical protein
			3032643	3033803	Phage protein
			3033864	3034262	hypothetical protein
			3034253	3034609	hypothetical protein
			3034727	3035158	hypothetical protein
			3035155	3036276	Chromosome (plasmid) partitioning protein ParB
			3036483	3036749	hypothetical protein
			3036762	3036881	hypothetical protein
			3037205	3037594	transcriptional regulator, XRE family
			3037704	3038891	Phage integrase
3757106	3769706	12600	3757082	3757198	hypothetical protein
			3757584	3757697	hypothetical protein
			3757782	3758432	hypothetical protein
			3758605	3758820	hypothetical protein
			3759272	3759421	hypothetical protein
			3760101	3761093	hypothetical protein
			3761099	3761932	CRISPR-associated RAMP Cmr6
			3761952	3762323	CRISPR-associated RAMP Cmr5
			3762320	3763279	CRISPR-associated RAMP Cmr4
			3763269	3764558	CRISPR-associated RAMP Cmr3
			3764527	3766347	CRISPR-associated RAMP Cmr2
			3766347	3767900	CRISPR-associated RAMP Cmr1
			3768679	3769359	hypothetical protein

3810554	3822720	12166	3810554	3810703	hypothetical protein
			3810954	3811577	Methyltransferase (EC 2.1.1.-)
			3811599	3812054	Conserved domain protein
			3812401	3813123	Methyltransferase (EC 2.1.1.-)
			3813133	3813276	Methyltransferase (EC 2.1.1.-)
			3813325	3813588	hypothetical protein
			3813910	3814350	hypothetical protein
			3814422	3815045	hypothetical protein
			3815065	3815643	hypothetical protein
			3817658	3818509	Possible aminoglycoside phosphotransferase
			3818503	3818952	hypothetical protein
			3819061	3819951	hypothetical protein
			3820090	3820536	hypothetical protein
			3820743	3821282	hypothetical protein
			3821470	3821883	Histone acetyltransferase HPA2
			3821973	3822422	GCN5-related N-acetyltransferase
			3822583	3822720	hypothetical protein
3829120	3842427	13307	3829120	3829629	hypothetical protein
			3829767	3830411	hypothetical protein
			3830585	3830797	hypothetical protein
			3831202	3831384	Ubiquinone/menaquinone biosynthesis methyltransferase UBIE (EC 2.1.1.-)
			3831398	3832015	Ubiquinone/menaquinone biosynthesis methyltransferase UBIE (EC 2.1.1.-)
			3832211	3832936	hypothetical protein
			3833938	3835179	hypothetical protein
			3835433	3836584	hypothetical protein
			3836656	3836934	hypothetical protein
			3837344	3838303	putative cytoplasmic protein
			3838463	3838951	hypothetical protein
			3839126	3839764	hypothetical protein
			3839983	3840186	hypothetical protein
			3840708	3841319	hypothetical protein
			3841454	3841681	Phage integrase
			3841678	3841854	hypothetical protein
			3842047	3842427	hypothetical protein
3962011	3972716	10705	3962011	3962316	hypothetical protein
			3962827	3964182	hypothetical protein
			3964336	3966060	ATPase involved in DNA repair
			3966265	3967248	hypothetical protein
			3967472	3968158	Sll1429 protein
			3968422	3968637	hypothetical protein
			3968686	3969729	ankyrin repeat protein, putative
			3970588	3971730	hypothetical protein
			3971865	3972716	hypothetical protein
4167992	4213959	45967	4168136	4173100	beta-ketoacyl synthase

			4173093	4180103	Malonyl CoA-acyl carrier protein transacylase (EC 2.3.1.39)
			4180118	4180237	hypothetical protein
			4180213	4181043	Chaperone protein DnaK
			4181262	4181924	Chaperone protein DnaK
			4182136	4189719	Malonyl CoA-acyl carrier protein transacylase (EC 2.3.1.39)
			4189761	4194407	Malonyl CoA-acyl carrier protein transacylase (EC 2.3.1.39)
			4194380	4197388	Malonyl CoA-acyl carrier protein transacylase (EC 2.3.1.39)
			4197385	4199682	Malonyl CoA-acyl carrier protein transacylase (EC 2.3.1.39)
			4199676	4199837	hypothetical protein
			4199936	4200079	hypothetical protein
			4200184	4201230	O-methyltransferase
			4201212	4201946	Aspartate racemase (EC 5.1.1.13)
			4202020	4205814	Peptide synthetase
			4205851	4209216	Malonyl CoA-acyl carrier protein transacylase (EC 2.3.1.39)
			4209473	4210084	Transcription antitermination protein NusG
			4210512	4211114	Transcriptional regulator, TetR family
			4211396	4213753	Phosphoenolpyruvate synthase (EC 2.7.9.2)
4310402	4318130	7728	4310402	4311934	Radical SAM domain protein
			4311900	4313573	ABC-type multidrug/protein/lipid transport system, ATPase component
			4313820	4314467	hypothetical protein
			4314461	4315789	Arylsulfatase regulator (Fe-S oxidoreductase)
			4316031	4316144	hypothetical protein
			4316275	4317111	response regulator
			4317117	4318130	two-component sensor histidine kinase
4821202	4855690	34488	4821202	4821432	hypothetical protein
			4821879	4823249	Bacitracin synthetase 3 (BA3)
			4823209	4832796	FIG01233961: hypothetical protein
			4833027	4834760	Lipid A export ATP-binding/permease protein MsbA
			4834757	4836583	Lipid A export ATP-binding/permease protein MsbA
			4836573	4839881	Siderophore biosynthesis non-ribosomal peptide synthetase modules
			4840036	4855029	hypothetical protein
			4855105	4855290	hypothetical protein
			4855577	4855690	hypothetical protein

Table S3. Genome features of members belonging to genus *Paenibacillus*

Sl no.	Name	Genome assembly ID	Location of isolation	Ecological occurrence	References	Genome size (Mb)	GC content (%)	Total Genes*	Total Proteins*
1.	*P. polymyxa* A18	NZ_JWJJ00000000.1	Termite gut, India	Gut	Current Study	5.72	46.2	5066	4,805
2.	*P. polymyxa* CF05	NZ_CP009909.1	Cryptomeria fortunei tree, China	Rhizosphere	1	5.76	45.5	4,928	4,715
3.	*P. polymyxa* SQR-21	NZ_CP006872.1	Watermelon rhizosphere	Rhizosphere	2	5.82	45.64	5,030	4,768
4.	*P. polymyxa* M1	NC_017542.1	Wheat roots, China	Rhizosphere		6.23	44.8	5,596	5,372
5.	*P. polymyxa* SC2	NC_014622.2	Pepper rhizosphere, Guizhou, China	Rhizosphere	3	6.24	44.58	5,747	5,474
6.	*P. polymyxa* Sb3-1	NZ_CP010268.1	Agricultural soil, Egypt	Rhizosphere	4	5.82	45.37	5,050	4,802
7.	*P. peoriae* HS311	NZ_CP011512.1	Korea	Rhizosphere	unpublished	6.01	45.7	5,507	5,365
8.	*P. polymyxa* strain ATCC 15970	NZ_CP011420.1	Soil, Belgium	Rhizosphere	unpublished	6.09	45.6	5,325	4,991
9.	*P. polymyxa* strain YC0573	NZ_CP017968.2	rhizosphere of tobacco, China	Rhizosphere	unpublished	6.12	45.6	5,334	5,194
10.	*P. polymyxa* strain J	NZ_CP015423.1	China	NA	unpublished	5.75	45.7	5,175	5,023
11.	*P. polymyxa* CR1	NC_023037.2	Corn roots, Canada	Rhizosphere	5	6.01	45.6	5,301	5,029
12.	*P. polymyxa* strain YC0136	NZ_CP017967.1	Rhizosphere of Tobacco, China	Rhizosphere	unpublished	5.62	45.7	4,937	4,808
13.	*P. polymyxa* E681	NC_014483.2	Barley rhizosphere, South Korea	Rhizosphere	6	5.39	45.8	4,796	4,528
14.	*P. terrae* HPL-003	NC_016641.1	Forest residue, Iraq	Rhizosphere	7	6.08	46.77	5,396	5,137
15.	*P. xylanexedens* strain PAMC 22703	NZ_CP018620.1	Sediments, Russia	Rhizosphere	unpublished	7.05	46	6,208	6,066
16.	*P. riograndensis* SBR5	NZ_LN831776	Wheat field, Brazil	Soil	unpublished	7.5	51	7,326	7,286
17.	*P. sp.* FSL H7-0357	NZ_CP009241.1	Pasteurized milk, USA	NA	unpublished	7.7	49.1	6,857	6,741
18.	*P. graminis*strain DSM 15220	NZ_CP009287.1	Maize rhizosphere, France	Rhizosphere	unpublished	7.16	50.6	5,763	5,584
19.	*P. sp.* FSL P4-0081	NZ_CP009280.1	heat treated raw milk, USA	NA	unpublished	8.42	51.1	7,302	7,183
20.	*P. sp.* FSL R7-0273	NZ_CP009283.1	Milk, USA	NA	unpublished	7.18	51.9	6,231	6,180

21.	*P. borealis* strain DSM 13188	NZ_CP009285.1	Spruce forest humus, Finland	NA	unpublished	8.15	51.4	6,382	6,213
22.	*P. odorifer* strain DSM 15391	NZ_CP009428.1	Radioactive waste repository, Finland	NA	unpublished	6.81	44.2	5,593	5,431
23.	*P. sp.* FSL R7-0331	NZ_CP009284.1	Pasteurized fat milk, USA	NA	unpublished	6.93	51.2	6,066	5,947
24.	*P. sp.* FSL R5-0912	NZ_CP009282.1	Pasteurized fat milk, USA	NA	unpublished	7.71	51.1	6,662	6,540
25.	*P. sp.* FSL H7-0737	NZ_CP009279.1	Pasteurized milk, USA	NA	unpublished	6.76	43.7	5,961	5,836
26.	*P. bovis sp.* BD3526	NZ_CP013023.1	Yak milk, China	NA	unpublished	5.35	47.3	4,567	4,450
27.	*P. sp.* Y412MC10	NC_013406.1	Hot Spring, Montana, USA	Intestine	8	7.12	51.2	6,302	6,152
28.	*P. sp.* FSL R5-0345	NZ_CP009281.1	Pasteurized fat milk, USA	NA	unpublished	6.78	43.7	6,037	5,914
29.	*P. glucanolyticus* strain 5162	NZ_CP015286.1	Soil	NA	unpublished	5.89	49.2	5,285	5,184
30.	*P. sp.* IHBB 10380	NZ_CP010976.1	Soil, India	NA	unpublished	5.77	41.29	5,313	5,185
31.	*P. durus* strain DSM 1735	NZ_CP009288.1	marine sediment	NA	unpublished	6.03	50.8	5,453	5,338
32.	*P. mucilaginosus* K02	NC_017672.3	Soil	NA	9	8.81	58.3	7,403	7,209
33.	*P. durus* ATCC 35681	NZ_CP011114.1	NA	NA	10	5.57	50.8	5,308	5,179
34.	*P. stellifer* strain DSM 14472	NZ_CP009286.1	Paperboard, Finland	NA	11	5.65	53.5	4,675	4,464
35.	*P. mucilaginosus* KNP414	NC_015690.1	Soil of Tianmu Mountain, China	Rhizosphere	12	8.66	58.38	7,173	6,909
36.	*P. sabinae* T27	NZ_CP004078.1	rhizosphere soil	Rhizosphere	13	5.27	52.6	4,904	4,792
37.	*P. mucilaginosus* 3016	NC_016935.1	rhizosphere soil	Rhizosphere	14	8.73	58.3	7,442	7,221
38.	*P. yonginensis* sp. DCY84	NZ_CP014167.1	humus soil, South Korea	NA	15	4.98	51	4,558	4,441
39.	*P. sp.* LPB0068	NZ_CP017770.1	Pacific oyster (Crassostreagigas), South Korea	NA	16	4.62	40	4,461	4,336
40.	*P. sp.* JDR-2	NC_012914.1	Sweet gum stem, USA	Rhizosphere	17	7.18	50.28	6,322	6,128
41.	*P. beijingensis* strain DSM 24997	NZ_CP011058.1	Jujube rhizosphere soil, China	Rhizosphere	18	5.74	52.5	5,215	4,728
42.	*P. swuensis* strain DY6	NZ_CP011388.1	Soil, South Korea	NA	19	5.01	48.9	4,551	4,430
43.	*P. sp.* 32O-W	NZ_CP013653.1	Soil, USA	NA	20	5.37	56.3	4,844	4,754

44.	*P. naphthalenovorans* strain 32O-Y	NZ_CP013652.1	Soil, USA	NA	20	5.2	49.7	5,135	5,004
45.	*P. larvae* subsp. pulvifaciens strain CCM 38	NZ_CP020327	NA	NA	NA	4.33	44.19	4,611	4,504
46.	*P. larvae* subsp. larvae DSM 25430	NC_023134.1	Honey bee broods,Tunisia	Honey bee pathogen	21	4.05	45	3,993	3,683
47.	*P. larvae* subsp. larvae strain ATCC 9545	NZ_CP019687	NA	Honey bee pathogen	NA	4.28	44.2	4,496	4,388
48.	*P. larvae* subsp. pulvifaciens strain ATCC 13537	NZ_CP019794	NA	NA	NA	4.41	44.19	4,778	4,671

NA-Not Available

*The total genes and proteins represented here are based on NCBI annotation

Table S4: Categorization of CAZy genes encoded in *P. polymyxa* A18 according to their functions

Enzyme	EC Number	CAZy Family	RAST ID	NCBI ID
**1.Cellulose-degrading enzymes**				
Cellobiohydrolase	3.2.1.14	GH6	fig|6666666.120009.peg.1002	WP_016821398.1
β -1,3-endoglucanase		GH81	fig|6666666.120009.peg.4288	WP_017425583.1
β-1,4-endoglucanase		GH5	fig|6666666.120009.peg.2467	WP_017426982.1
	3.2.1.4	GH5	fig|6666666.120009.peg.5008	WP_038978163.1
	3.2.1.4	GH5	fig|6666666.120009.peg.3617	WP_017426793.1
		GH5	fig|6666666.120009.peg.3481	WP_017428294.1
		GH48	fig|6666666.120009.peg.2298	
	3.2.1.3	GH26	fig|6666666.120009.peg.4472	WP_017427072.1
	3.2.1.4	GH44	fig|6666666.120009.peg.4472	WP_017427072.1
Xyloglucan-specific β-1,4-endoglucanase		GH74	fig|6666666.120009.peg.2569	WP_017427741.1
β-1,3(4)-endoglucanase	3.2.1.73	GH16	fig|6666666.120009.peg.2386	WP_017426919.1
β-glucosidase	3.2.1.21	GH1	fig|6666666.120009.peg.1624	WP_017425890.1
	3.2.1.21	GH1	fig|6666666.120009.peg.3835	WP_026065451.1
	3.2.1.21	GH1	fig|6666666.120009.peg.3948	WP_017427334.1
	3.2.1.21	GH1	fig|6666666.120009.peg.4226	WP_017425532.1
	3.2.1.21	GH1	fig|6666666.120009.peg.4845	WP_026065363.1
	3.2.1.21	GH3	fig|6666666.120009.peg.903	WP_017426284.1
	3.2.1.21	GH3	fig|6666666.120009.peg.1068	WP_017426395.1

	3.2.1.21	GH3	fig|6666666.120009.peg.2390	WP_038978010.1
	3.2.1.21	GH3	fig|6666666.120009.peg.2427	WP_017426950.1
	3.2.1.21	GH3	fig|6666666.120009.peg.285	WP_038978231.1
	3.2.1.21	GH3	fig|6666666.120009.peg.3484	WP_017428297.1
	3.2.1.21	GH3	fig|6666666.120009.peg.4693	WP_017428582.1
**2. Hemicellulose-degrading enzymes**				
α-galactosidase	3.2.1.22	GH27	fig|6666666.120009.peg.2230	WP_017428506.1
	3.2.1.22	GH36	fig|6666666.120009.peg.2321	WP_017426877.1
	3.2.1.22	GH36	fig|6666666.120009.peg.2477	WP_016820825.1
	3.2.1.22	GH36	fig|6666666.120009.peg.516	WP_017428015.1
	3.2.1.22	GH36	fig|6666666.120009.peg.691	WP_016819342.1
	3.2.1.22	GH4	fig|6666666.120009.peg.1001	WP_016821399.1
	3.2.1.22	GH4	fig|6666666.120009.peg.964	WP_026065381.1
β-1,4-endoxylanase	3.2.1.8	GH5	fig|6666666.120009.peg.3656	WP_026065408.1
	3.2.1.8	GH5	fig|6666666.120009.peg.2343	WP_016818773.1
		GH10	fig|6666666.120009.peg.4954	WP_013373834.1
	3.2.1.8	GH10	fig|6666666.120009.peg.4797	WP_017426140.1
		GH11	fig|6666666.120009.peg.4323	WP_017425612.1
		GH30	fig|6666666.120009.peg.610	WP_017425752.1
	3.2.1.8	GH43	fig|6666666.120009.peg.2385	WP_017426918.1
β-1,4-xylosidase	3.2.1.37	GH43	fig|6666666.120009.peg.3200	WP_017428110.1
		GH43	fig|6666666.120009.peg.4843	WP_017426115.1
	3.2.1.37	GH52	fig|6666666.120009.peg.4822	WP_017426124.1
α-arabinofuranosidase		GH43	fig|6666666.120009.peg.269	WP_017425677.1
	3.2.1.55	GH43	fig|6666666.120009.peg.1753	WP_017425805.1
	3.2.1.55	GH51	fig|6666666.120009.peg.188	WP_038978238.1
	3.2.1.55	GH51	fig|6666666.120009.peg.296	WP_038978229.1
	3.2.1.55	GH51	fig|6666666.120009.peg.3030	WP_017427480.1
α -1,5-endoarabinosidase	3.2.1.99	GH43	fig|6666666.120009.peg.796	WP_017426194.1
	3.2.1.99	GH43	fig|6666666.120009.peg.1752	WP_017425806.1
Mannanase		GH5	fig|6666666.120009.peg.576	WP_017427981.1
β-1,4-mannosidase	3.2.1.78	GH26	fig|6666666.120009.peg.522	WP_017428011.1
	3.2.1.78	GH26	fig|6666666.120009.peg.635	WP_017425735.1
	3.2.1.78	GH26	fig|6666666.120009.peg.3186	WP_017428102.1
		GH26	fig|6666666.120009.peg.1157	WP_017426461.1
α-1,5-exoarabinanase/endo-β-1,4-galactanase		GH53	fig|6666666.120009.peg.3141	WP_017427835.1
Polygalacturonase	3.2.1.15	GH28	fig|6666666.120009.peg.3203	WP_017428113.1
Acetyl xylan esterase		CE1	fig|6666666.120009.peg.4249	WP_017425551.1
		CE1	fig|6666666.120009.peg.4482	WP_017427076.1
xylanase deacetylase	3.5.1.-	CE4	fig|6666666.120009.peg.2340	WP_017426884.1
		CE8	fig|6666666.120009.peg.4307	WP_017425600.1
**3. Pectin-degrading enzymes**				

Pectinesterase			fig|6666666.120009.peg.3201	WP_017428111.1
Pectin lyase	4.2.2.10	PL1	fig|6666666.120009.peg.1417	WP_017426635.1
Pectate lyase	4.2.2.2	PL1	fig|6666666.120009.peg.3626	WP_016821905.1
	4.2.2.2	PL1	fig|6666666.120009.peg.4834	WP_017426120.1
	4.2.2.2	PL1	fig|6666666.120009.peg.4834	WP_017426120.1
	4.2.2.2	PL1	fig|6666666.120009.peg.210	WP_017425629.1
		PL10	fig|6666666.120009.peg.1582	WP_017425913.1
		PL3	fig|6666666.120009.peg.870	WP_016819868.1
		PL9	fig|6666666.120009.peg.2463	WP_017426980.1
Rhamnogalacturonanendolyase		PL11	fig|6666666.120009.peg.1582	WP_017425913.1
		PL11	fig|6666666.120009.peg.985	WP_017426343.1
		PL9	fig|6666666.120009.peg.2463	WP_017426980.1
		PL27	fig|6666666.120009.peg.963	WP_038978105.1
α-rhamnosidase	3.2.1.40	GH78	fig|6666666.120009.peg.3499	WP_017428310.1
		GH106	fig|6666666.120009.peg.2393	WP_038978012.1
Endo-apiosidase	3.2.1.-	GH140	fig|6666666.120009.peg.3943	WP_026065445.1
		GH140	fig|6666666.120009.peg.4110	WP_017428672.1
α-L-fucosidase	3.2.1.51	GH141	fig|6666666.120009.peg.3588	WP_017426818.1

Table S5: Annotation of antibiotic synthesizing polypeptides in *P. polymyxa* A18

**Antibiotics synthesized**	**Protein involved in synthesis**	**NCBI Protein ID**
Polymyxin	Polymyxin synthetase A, pmx A	WP_017427759.1
	Polymyxin synthetase B, pmxB	WP_038978210.1
	Polymyxin synthetase C, pmxC, transporter	WP_017428090.1
	Polymyxin synthetase D, pmxD, transporter	WP_017428091.1
	Polymyxin synthetase E, pmx E	fig|6666666.120009.CDS.4336
Paenibacillin	PaeA, paenibacillin prepropeptide	
	PaeP, peptidase	WP_016819874.1
	PaeB, lantibiotic dehydratase	WP_038978102.1 52..667
	PaeC, lantibiotic cyclase	WP_017426262.1 46..419
	PaeI, putative immunity protein	
	PaeT, transporter	WP_026065373.1
	AgrB, accessory gene regulator B, processing signal molecule	WP_019686238.1
	AgrD, autoinducing peptide (AIP)	fig|6666666.120009.CDS.881
	AgrC, histidine kinase	WP_017426266.1
	AgrA, response regulator	WP_017426267.1
	PaeN, putative acetylase	WP_017426268.1
Tridecaptin	TrbA thioestrase	WP_017428487.1
	TrbB ABC transporter	WP_038978247.1

	TrbC ABC transporter	WP_017428489.1
	TrbD NRPS	WP_038978246.1
	TrbE NRPS	WP_026065510.1
Fusaricidin	FusA	WP_017427039.1
	FusB	WP_017427040.1
	FusC	WP_016818887.1
	FusD	WP_017427041.1
	FusE	WP_017427883.1
	FusF	WP_017427042.1
	FusG	WP_017427043.1

Table S6. Concentration of antibiotics used for susceptibility testing

**Antibiotic tested**	**Range (µg/ml)**	**Concentration used for testing (µg/ml)**
Tetracycline	1-150	1, 2.5, 5, 10, 15, 20, 30, 40, 50, 60, 70, 80, 90, 100, 110, 120, 130, 140, 150
Erythromycin	1-25	1, 2.5, 5, 10, 15, 20, 25
Kanamycin	10-150	10, 20, 30, 40, 50, 60, 70, 80, 90, 100, 110, 120, 130, 140, 150
Chloramphenicol	10-150	10, 20, 30, 40, 50, 60, 70, 80, 90, 100, 110, 120, 130, 140, 150
Penicillin	10-100	10, 20, 30, 40, 50, 60, 70, 80, 90, 100
Vancomycin	10-90	10, 20, 30, 40, 50, 60, 70, 80, 90

References

1 Lei, M. *et al.* Complete genome sequence of Paenibacillus polymyxa CF05, a strain of plant growth-promoting rhizobacterium with elicitation of induced systemic resistance. *Genome announcements* **3**, e00198-00115 (2015).

2 Li, S. *et al.* Complete genome sequence of Paenibacillus polymyxa SQR-21, a plant growth-promoting rhizobacterium with antifungal activity and rhizosphere colonization ability. *Genome announcements* **2**, e00281-00214 (2014).

3 Ma, M. *et al.* Complete genome sequence of Paenibacillus polymyxa SC2, a strain of plant growth-promoting Rhizobacterium with broad-spectrum antimicrobial activity. *Journal of bacteriology* **193**, 311-312, doi:10.1128/JB.01234-10 (2011).

4 Rybakova, D., Wetzlinger, U., Müller, H. & Berg, G. Complete genome sequence of Paenibacillus polymyxa strain SB3-1, a soilborne bacterium with antagonistic activity toward plant pathogens. *Genome announcements* **3**, e00052-00015 (2015).

5 Weselowski, B., Nathoo, N., Eastman, A. W., MacDonald, J. & Yuan, Z.-C. Isolation, identification and characterization of Paenibacillus polymyxa CR1 with potentials for biopesticide, biofertilization, biomass degradation and biofuel production. *BMC microbiology* **16**, 244 (2016).
6 Kim, J. F. *et al.* Genome sequence of the polymyxin-producing plant-probiotic rhizobacterium Paenibacillus polymyxa E681. *Journal of bacteriology* **192**, 6103-6104 (2010).
7 Shin, S. H. *et al.* Genome sequence of Paenibacillus terrae HPL-003, a xylanase-producing bacterium isolated from soil found in forest residue. *Journal of bacteriology* **194**, 1266, doi:10.1128/JB.06668-11 (2012).
8 Mead, D. A. *et al.* Complete genome sequence of Paenibacillus strain Y4. 12MC10, a novel Paenibacillus lautus strain isolated from Obsidian Hot Spring in Yellowstone National Park. *Standards in genomic sciences* **6**, 366 (2012).
9 Xiao, B., Sun, Y.-F., Lian, B. & Chen, T.-M. Complete genome sequence and comparative genome analysis of the Paenibacillus mucilaginosus K02. *Microbial pathogenesis* **93**, 194-203 (2016).
10 Halim, M. A. *et al.* Genome sequence of a Gram-positive diazotroph, Paenibacillus durus type strain ATCC 35681. *Genome announcements* **4** (2016).
11 Suominen, I. *et al.* Paenibacillus stellifer sp. nov., a cyclodextrin-producing species isolated from paperboard. *International journal of systematic and evolutionary microbiology* **53**, 1369-1374 (2003).
12 Hu, X., Chen, J. & Guo, J. Two phosphate-and potassium-solubilizing bacteria isolated from Tianmu Mountain, Zhejiang, China. *World journal of Microbiology and Biotechnology* **22**, 983-990 (2006).
13 Ma, Y., Xia, Z., Liu, X. & Chen, S. Paenibacillus sabinae sp. nov., a nitrogen-fixing species isolated from the rhizosphere soils of shrubs. *International journal of systematic and evolutionary microbiology* **57**, 6-11 (2007).
14 Ma, M. *et al.* Complete genome sequence of Paenibacillus mucilaginosus 3016, a bacterium functional as microbial fertilizer. *Journal of bacteriology* **194**, 2777-2778 (2012).
15 Sukweenadhi, J. *et al.* Paenibacillus yonginensis DCY84 T induces changes in Arabidopsis thaliana gene expression against aluminum, drought, and salt stress. *Microbiological research* **172**, 7-15 (2015).
16 Shin, S.-K., Kim, E. & Yi, H. Paenibacillus crassostreae sp. nov., isolated from the Pacific oyster Crassostrea gigas. *International Journal of Systematic and Evolutionary Microbiology* (2017).
17 Chow, V. *et al.* Complete genome sequence of Paenibacillus sp. strain JDR-2. *Standards in genomic sciences* **6**, 1 (2012).
18 Kwak, Y. & Shin, J.-H. Complete genome sequence of Paenibacillus beijingensis 7188 T (= DSM 24997 T), a novel rhizobacterium from jujube garden soil. *Journal of biotechnology* **206**, 75-76 (2015).
19 Lee, J.-J. *et al.* Paenibacillus swuensis sp. nov., a bacterium isolated from soil. *The Journal of Microbiology* **52**, 106 (2014).
20 Butler, R. R., Wang, J., Stark, B. C. & Pombert, J.-F. Complete genome sequences of two interactive moderate thermophiles, Paenibacillus napthalenovorans 32O-Y and Paenibacillus sp. 32O-W. *Genome announcements* **4**, e01717-01715 (2016).
21 Djukic, M. *et al.* How to kill the honey bee larva: genomic potential and virulence mechanisms of Paenibacillus larvae. *PloS one* **9**, e90914 (2014).
